# Sustained drug-releasing hydrogel coatings for ureteral stents to prevent iatrogenic injury-induced ureteral stricture

**DOI:** 10.1016/j.mtbio.2025.102481

**Published:** 2025-10-28

**Authors:** Dawei Tian, Jian Wang, Guoqiang Han, Weiwei Wang, Lei Cui, Chenning Li, Wei Wang, Zhenhua Yang, Diansheng Zhou, Xiepeng Zuo, Zesheng An, Tianxiao Xu, Jian Zhu

**Affiliations:** a Department of Urology, Tianjin Institute of Urology, The Second Hospital of Tianjin Medical University, Tianjin, 300202, PR China; b School of Materials Science and Engineering, National Institute for Advanced Materials, Smart Sensing Interdisciplinary Science Center, Nankai University, Tianjin, 300350, PR China

**Keywords:** Hydrogel coating, Nanoparticles, Pirfenidone, Ureteral stent, Ureteral stricture

## Abstract

Iatrogenic injury-induced ureteral stricture often occurs during the treatment of ureteral disorders, and can lead to serious consequences requiring further medical care. The stricture prevention requires effective strategies to inhibit fibrosis, reduce oxidative stress and inflammatory responses during the insertion of ureteral stents. Here, we explore the anti-stricture effects of commercial ureteral stents modified with hydrogel coatings, which contain functionalized poly (lactic-co-glycolic acid) (fPLGA) nanoparticles (NPs) loaded with the drug of pirfenidone (PFD). The polyacrylamide (PAM) hydrogel coatings are robustly grafted onto the polyurethane (PU) stent using the surface initiated radical polymerization, while PFD-fPLGA NPs decorated with acryloyl functional groups were securely anchored in PAM networks during the reaction. This NPs-hydrogel coating possesses multiple advantages, such as high hydrophilicity and low elastic modulus, which can reduce frictional force and alleviate irritation during stent insertion. The hydrogel coating exhibits excellent stability, with only 45 % mass loss after being immersed in an artificial urine environment for 3 months, and also demonstrates good biocompatibility and antifouling performance. More importantly, the slow degradation of fPLGA leads to sustained delivery of antifibrotic PFD, with a cumulative drug release rate of approximately 82 % within 12 weeks. These combined benefits contribute to the prevention of ureteral strictures after iatrogenic injury, as evidenced by inhibited fibrosis, alleviated oxidative stress, and suppressed inflammatory response in both vitro and in vivo studies.

## Highlights

• A sustained drug-releasing ureteral stent to prevent ureteral stricture.
• Nanoparticles-hydrogel coatings prepared have excellent overall properties.
• The functional coatings can ensure a sustained drug-releasing period to 12 weeks.
• The stent successfully inhibited ureteral stricture by reducing fibrosis, inflammation and oxidative stress.

## Graphical abstract

Here, we explore the anti-stricture effects of commercial ureteral stents modified with hydrogel coatings, which contain PFD-fPLGA NPs. The hydrogel coatings are robustly grafted onto the stent, while PFD-fPLGA NPs were securely anchored in PAM networks during the reaction. Such NPs-hydrogel coated ureteral stents demonstrate sustained-release performance high biocompatibility and antifouling capability. These combined benefits contribute to the prevention of ureteral strictures after iatrogenic injury. Overall, our research developed a drug sustained-release delivery platform with great potential for clinical application.

**Figure:**
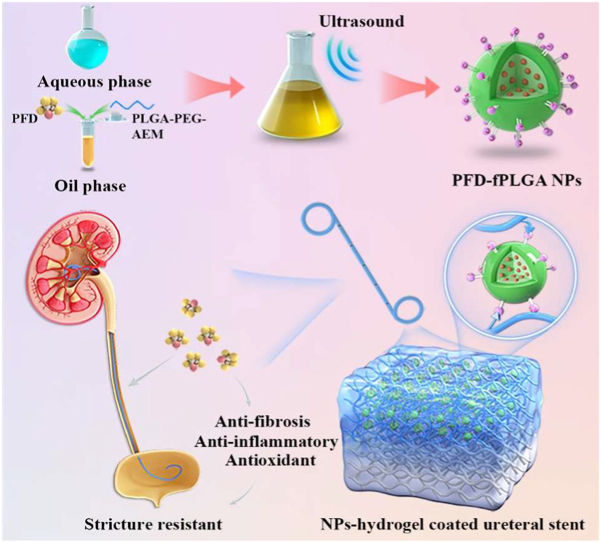

## 1 Introduction

Ureteral stricture (US) is a prevalent urological disease marked by abnormally narrowed ureteral lumen [1,2]. Such condition is frequently attributed to inflammation, calculi, trauma, neoplasms, and iatrogenic injury, which can result in varying degrees of upper urinary tract obstruction and subsequent hydronephrosis [3,4]. The growing use of endoscopic techniques in recent clinical practice has resulted in a higher incidence of iatrogenic US. In particular, the occurrence of US in patients with ureteral stones following endoscopic treatment can reach up to 5 %, which is associated with local inflammatory responses, laser-induced thermal injury, mechanical irritation from stones, and compression of the ureteral mucosa [5,6]. Ureteral injury triggers inflammation and oxidative stress, which together promote excessive fibrosis and tissue remodeling, ultimately leading to US [[7], [8], [9], [10]]. US can lead to severe complications and potentially life-threatening conditions, imposing substantial burdens on patients. Severe US would require surgical treatment which is usually fraught with medical risks, potential complications and high costs [11,12]. Therefore, effectively preventing and managing iatrogenic US represents a critical challenge [13].

Recently, ureteral stents with drug-releasing capabilities have been used to prevent the iatrogenic US [[14], [15], [16], [17], [18]]. A useful stent would support drainage, delay the progression of stricture, and promote tissue repair, when it is inserted into ureter with iatrogenic injury [15,19,20]. In comparison, traditional ureteral stent is unable to heal ureteral surface wounds, and can cause lower urinary tract symptoms, infection, and secondary stricture [[21], [22], [23], [24], [25], [26]]. The effective drugs, such as pirfenidone (PFD), paclitaxel and rapamycin, are usually antifibrotic, and can promote the wound healing by suppressing excessive tissue fibrosis or scarring [[27], [28], [29], [30], [31], [32], [33], [34]]. These drugs need to be released slowly, as the limited blood supply to the ureteral mucosa and constant urine flow significantly extend the mucosal healing period to approximately 6 weeks [[35], [36], [37]]. Current sustained-release strategies rely on the physical embedding of drugs or drug-loaded nanoparticles (NPs) within functional polymer layers coated on ureteral stent walls [15,16,38]. However, the harsh ureteral environment can cause premature release of these drug layers, while friction during stent insertion may damage the coatings, limiting their suitability for comprehensive in vivo animal studies [14,21,39].

Hydrogels, as three-dimensional polymer networks characterized by high water content and excellent biocompatibility, have been widely explored in biomedical applications. However, their inherent porosity and hydrophilic nature often result in suboptimal controlled drug release kinetics [[40], [41], [42], [43]]. Conversely, NPs demonstrate superior drug release modulation but exhibit limited retention at target sites due to rapid clearance mechanisms. To address these limitations, an integrated approach combining these two drug delivery systems has emerged as a promising strategy. By engineering a hybrid NP-hydrogel system through physicochemical modifications, the complementary advantages of both systems can be leveraged—NPs enable sustained drug release, while hydrogels provide localized retention and enhance biocompatibility. This combinatorial strategy not only extends therapeutic efficacy but also minimizes systemic side effects by maintaining drug concentrations within therapeutically relevant ranges at the target site. Consequently, NP-hydrogel systems have garnered significant interest in recent years, with demonstrated utility in diverse applications such as localized therapy, tissue engineering, and regenerative medicine [[44], [45], [46], [47], [48]].

**Scheme 1:**
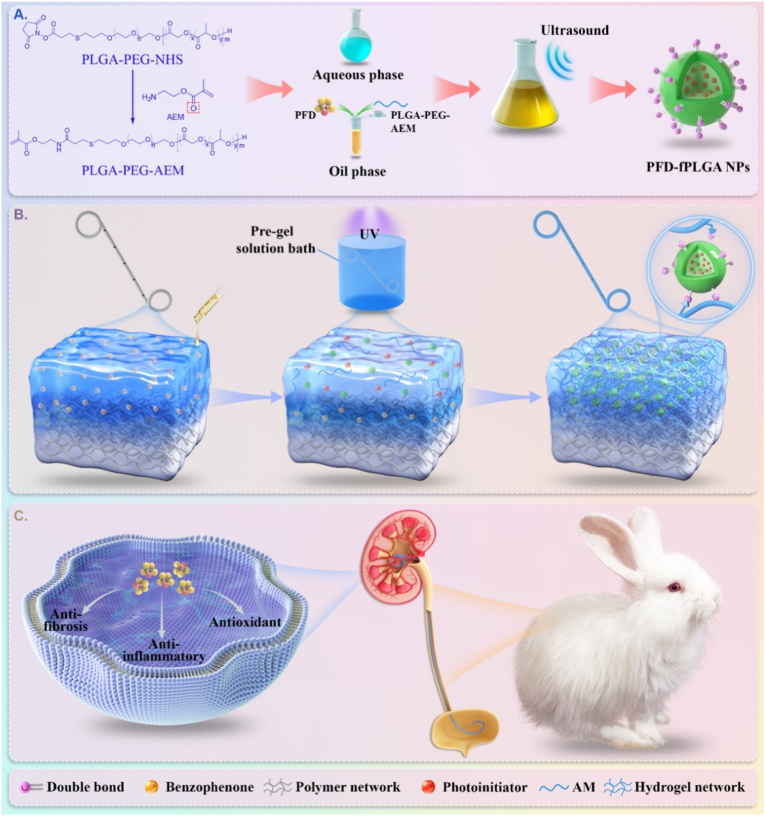
Schematic illustration of the fabrication of NPs-hydrogel coated ureteral stent for stricture-resistant. a) Modification of PLGA-PEG-NHS and preparation of PFD-fPLGA NPs. b) Preparation process of NPs-hydrogel coated ureteral stent. c) Effective mechanism of NPs-hydrogel coated ureteral stent: PFD is slowly released from the NPs-hydrogel system, exerting anti-fibrotic, anti-oxidative stress and anti-inflammatory effects, and ultimately inhibiting the occurrence and development of ureteral stricture.

Here, we present a sustained drug-releasing strategy (Scheme 1) for ureteral stent by exploiting robust polyacrylamide (PAM) hydrogel coatings containing PFD-loaded NPs to ultimately prevent iatrogenic US. The NPs have a matrix of functionalized poly (lactic-co-glycolic acid) (fPLGA), which contain acryloyl functional groups, for covalently binding into the hydrogel networks (Scheme 1a). In order to ensure a strong interfacial robustness between coatings and PU stents, PAM hydrogels are in-situ synthesized under UV light by leveraging the surface initiated radical polymerization approach [49,50], and PFD-fPLGA NPs are securely anchored in PAM networks during the reaction (Scheme 1b). Such robust and low friction functional coatings are highly biocompatible, and can ensure a sustained drug-releasing period to 12 weeks, leading to high in-vitro efficacy in reducing fibrosis, inflammation, and oxidative stress levels. Furthermore, such strategy proves to be effective in a rabbit ureteral injury model, and demonstrates a strong ability to promote wound repair and prevent stricture (Scheme 1c). Therefore, the NPs-hydrogel coated ureteral stent represents an ideal strategy for the prevention of iatrogenic US.

## 2 Results and discussion

### 2.1 Characterization of PFD-fPLGA NPs

To prepare the NPs-hydrogel coated ureteral stent for sustained drug release, fPLGA with acryloyl end groups were selected as the matrix of drug carrier to make their NPs chemically tetherable into hydrogel networks during radical polymerization reaction. fPLGA was synthesized through the condensation reaction between N-hydroxysuccinimide activated PLGA and 2-aminoethyl methacrylate (AEM). The NMR spectrum exhibited the anticipated proton chemical shifts, suggesting the successful synthesis of fPLGA (Fig. S1). Due to the addition of an excess amount of AEM, the double bond modification rate of the fPLGA is approximately 90.45 %.

**Fig. 1:**
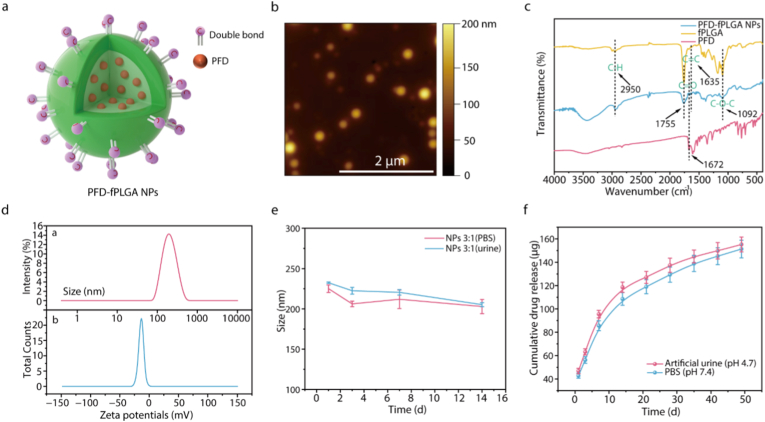
Characterization of PFD-fPLGA NPs. a) Schematic diagram of the structure of PFD-fPLGA NPs. b) Representative AFM image of PFD-fPLGA NPs. c) FTIR spectra of PFD, fPLGA and PFD-fPLGA NPs. d) Particle size and zeta potential distribution of PFD-fPLGA NPs (fPLGA: PFD 3:1). e) Stability experiments of NPs (fPLGA: PFD 3:1) in PBS and artificial urine. f) In vitro cumulative release curve of PFD-fPLGA NPs (fPLGA: PFD 3:1). (mean ± SD, n = 3).

PFD-fPLGA NPs were fabricated via a water-in-oil-in-water (W/O/W) double-emulsion solvent-evaporation method. During emulsification, the anti-stricture drug PFD was encapsulated within the PLGA matrix, with polyvinyl alcohol (PVA) employed as both emulsifier and steric stabilizer. Fig. 1a shows a schematic diagram of the PFD-fPLGA NPs. The surface of the NPs is grafted with carbon-carbon double bonds available for subsequent reactions, while the drugs are randomly dispersed in the polymer matrix. TEM and AFM images showed that the PFD-fPLGA NPs are spherical with a uniform particle size and a diameter of 214.60 ± 22.44 nm (Fig. 1b; Fig. S2), agreeing with the diameter estimated from light scattering measurement (Fig. 1d). The FTIR spectra of PFD-fPLGA NPs was shown in Fig. 1c. The characteristic C=O stretch at 1755 cm^−1^ from PLGA and C=O stretch from PFD pyridine ring at 1672 cm^−1^ confirmed the successful encapsulation of PFD in fPLGA matrix. The PFD-fPLGA NPs exhibited zeta potentials ranging from −18.8 mV to −15.2 mV, depending on the initial fPLGA: PFD ratio (Table S1, Fig. 1d and Fig. S3). The observed negative zeta potential is attributed to the exposure of carboxylic acid groups, likely resulting from partial hydrolysis of the ester bonds in fPLGA. We compared the in vitro drug release curves of different proportions of NPs. The results showed that the drug release performance of the fPLGA:PFD ratio of 3:1 was the best. The fPLGA:PFD ratios of 1:1 and 2:1 had excessive drug burst release in the early stage, while the fPLGA:PFD ratio of 5:1 had too slow early drug release amount and rate (Fig. S4). Since the fPLGA:PFD ratio of 3:1 achieved the optimal balance between encapsulation efficiency and drug loading capacity, this formulation was ultimately selected for the subsequent research (Table S1). The degradation and drug release profiles of PFD-fPLGA NPs were assessed in PBS solution and artificial urine. Under both conditions, the NPs size gradually decreased over time, indicating progressive degradation (Fig. 1e; Fig. S5). Correspondingly, PFD was slowly released into the surrounding media during the research period, with a cumulative release of approximately 75 % by the 7th week. Notably, drug release was accelerated in the more acidic artificial urine environment (Fig. 1f), likely due to enhanced hydrolysis of ester bonds under acidic conditions.

### 2.2 Characterization of NPs-hydrogel coated ureteral stent

**Fig. 2:**
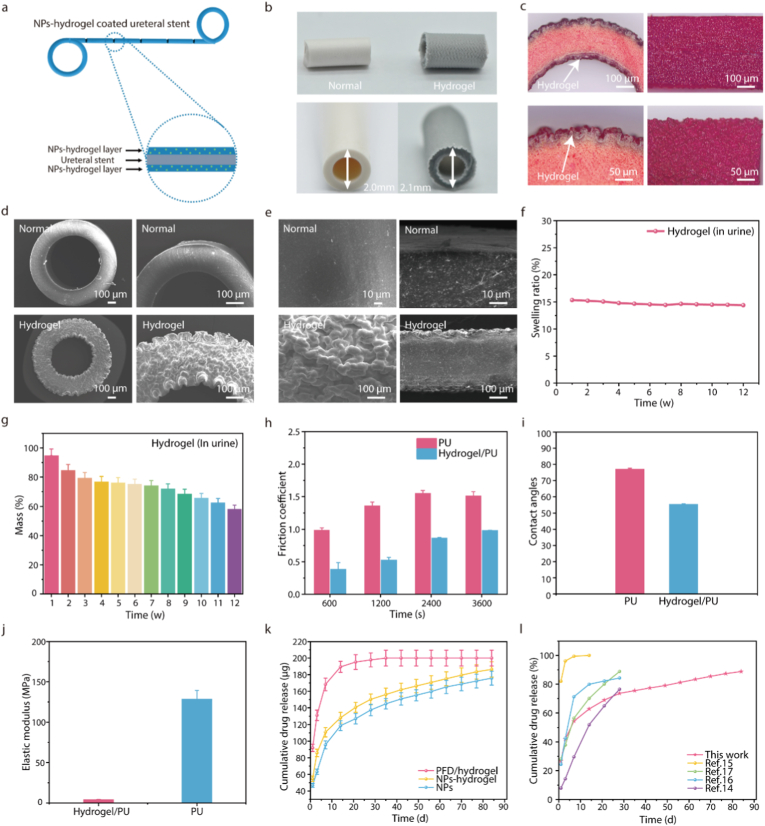
Characterization of NPs-hydrogel coated ureteral stent. a) Schematic diagram of the structure of the NPs-hydrogel coated ureteral stent. b) Camera images of the NPs-hydrogel coated ureteral stent and the normal ureteral stent. c) Optical microscope images of the NPs-hydrogel coated ureteral stent after staining. d, e) SEM images of the NPs-hydrogel coated ureteral stent and the normal stent. f) Swelling ratio. g) Mass loss. h) Friction coefficient. i) Water contact angle. j) Elastic modulus. k) In vitro cumulative release curves of the NPs-hydrogel coated ureteral stent. l) Comparison of cumulative release rate with the data reported in literature. (mean ± SD, n = 3).

To facilitate the coating of PFD-fPLGA NPs onto the ureteral stent, the stent was first immersed in a Benzophenone (BP) solution to enable thorough adsorption of the photo initiator onto its surface. It was subsequently transferred into a stable dispersion containing PFD-fPLGA NPs and acrylamide monomers. Upon UV irradiation, surface-initiated radical polymerization occurred [49,50], resulting in the formation of a hydrogel coating embedded with PFD-fPLGA NPs on the stent surface (Fig. 2a). Through the synergistic effect of the hydrophobic initiator and the hydrophilic initiator, a hydrogel-polymer interfacial interpenetrating network (IPN) is formed on the polymer surface. It was found that both inner and outer surfaces of the stent were covered with hydrogel coatings owing to their exposure to the UV light. When BP is exposed to ultraviolet radiation, a hydrogen abstraction reaction occurs, generating phenylpropenyl radicals and phenylpropenoyl imide derivatives. These molecules with conjugated double bonds cause the overall color of the stent to deepen, presenting a light yellow to light black color (Fig. S6). The white hydrogel coating could be seen on the surface (Fig. 2b). The hydrogel coatings became clearly visible under an optical microscope when stained with a red dye (Fig. 2c), and they exhibited a wavy bulging morphology, attributed to the swelling of the underlying polyurethane substrates. The hydrogel surface was dense and lacked apparent pores, a structure that can effectively inhibit the rapid release of the encapsulated materials (Fig. 2c–e). The hydrogel thickness was approximately 15 μm, and can be tunable by adjusting the concentrations of acrylamide monomers, ranging from 5 to 25 μm (Fig. S7). The porosity of the hydrogel coating is 45.65 % ± 2.34 %, and the average pore size is 16.84 ± 8.94 μm. The spatial distribution of Nile Red-labeled PFD-PLGA NPs confirms that the NPs were successfully and uniformly loaded into the hydrogel coating (Fig. S8).

In practical applications, the ureteral stents are exposed to complex environments, and the stability of the hydrogel coating is crucial for the sustained release of the drug. We evaluated the stability of the coating by measuring the swelling ratio and degradation rate of the hydrogel coating in artificial urine (Fig. 2f and g). The hydrogel coating exhibited long-term stability in the weakly acidic environment of artificial urine, with negligible swelling rate changes within three months and a mass loss of only about 45 %. Despite the quality of the hydrogel coating significantly declined, its morphology and structure remained stable for up to 4 months. We used a camera to record the changes on the surface of the stent at different time points and found that after being immersed in artificial urine for two months, there was no obvious change in the surface morphology of the stent (Fig. S9). However, by 6 months, the hydrogel coating exhibited noticeable changes and evident degradation (Fig. S10). This degradation rate is well-matched to the drug release requirements of the stent, thereby facilitating controlled and sustained drug release. We characterized the interfacial adhesion strength between the hydrogel coating and the stent substrate through tensile tests and scratch tests (Figure S11; Figure S11). The hydrogel coating could withstand large deformation (tensile ratio exceeding 4 times) without peeling off. Even when the PU stent fractured, the robust hydrogel-elastomer bond remained intact. In the steel needle scratch test, no obvious damage to the hydrogel coating was observed after 20 s of continuous scratching. This strong interfacial adhesion ensures the stability of the coating during stent insertion and in vivo retention.

Hydrogel coating can provide a highly lubricious and hydrophilic surface on the surface of stent. We measured the changes in the friction coefficient to evaluate the long-term mechanical robustness on PU films and PU films with hydrogel coating (0–3600 s at 3 KPa pressure). The presence of the hydrogel coating provided a significantly lower friction coefficient than PU films (Fig. 2h). The hydrogel coating also reduces the water contact angles of PU films and enhances the hydrophilicity of the material (Fig. 2i). Subsequently, we conducted an evaluation of the mechanical properties of the hydrogel surface to evaluate its ability to introduce a soft tissue-like surface on the polymer substrate. Surface elastic modulus measurements of the PU films and the PU films with hydrogel coating (15 μm thick) by AFM nanoindentation indicated that the presence of the hydrogel coating provided a low Young's modulus (E = 3.82 ± 0.56 MPa), which was significantly lower than the PU films (*E* = 128.34 ± 10.78 MPa) (Fig. 2j). Considering that the hydrogel coating is only present on the surface of the polymer substrate, the introduction of the hydrogel coating does not change the elastic modulus of the substrate (PU, 1 mm thick). The excellent mechanical strength of the hydrogel coating can be attributed to its unique hydrogel-polymer interpenetrating network structure, which significantly improves the mechanical robustness of the material through the interpenetration of the substrate and the hydrogel network.

In addition to excellent stability and mechanical properties, the hydrogel coating provides good anti-fouling properties to the surface of the stents. In order to quantitatively evaluate the antifouling performance of the hydrogel coating, the density of E. coli adhered to the surface of the hydrogel was compared and analyzed. The results showed that the level of E. coli adhesion on the hydrogel coating was significantly lower than that on the PU films (Fig. S13). This effective resistance to bacterial adhesion helps to delay the formation of biofilms on the surface of the stent, while enhancing the anti-infection performance of the stent. This is of great significance for medical devices implanted in the human body.

### 2.3 Drug release profile of the NPs-hydrogel coated ureteral stent in vitro

As shown in Fig. 2k and l, the release curves of PFD in NPs-hydrogel coated ureteral stent (Length, 1 cm) were determined in vitro. The results showed that the physical encapsulation of PFD in hydrogel coated stent leads to the fastest drug release, with over 90 % of the drug released cumulatively within 3 days. The release profiles of NPs-hydrogel coated stent and pure NPs exhibit similar release curves and the results show two phases. In the initial phase, rapid drug release is observed within the first 2 weeks, achieving a cumulative release of PFD up to 62 %. During the subsequent phase, the release amount decreased sharply within the next 10 weeks and the release rate declining markedly over time. The formation of this biphasic release mechanism can be attributed to the degradation characteristics of PLGA NPs and the regulatory effect of the hydrogel carrier. The early rapid release curve is mainly caused by the free drug adsorbed on the surface of the NPs and the drug released from the degradation of the surface layer of the NPs. The sustained and slow drug release in the later stage is due to the gradual hydrolysis of PLGA. The degradation period of the hydrogel coating was slower than the rate of drug release of PFD-fPLGA NPs, allowing the NPs to be completely released during this period. These results indicated that the NPs-hydrogel coated ureteral stent can release a large amount of PFD in the early stage to meet the acute phase demand, and then slowly release PFD over time to maintain long-term continuous treatment. Compared with previous studies, the in vitro release curve in this study is superior. It releases a sufficient number of drugs in the early stage to promote wound repair and continuously releases drugs in the later stage to inhibit ureteral stricture (Fig. 2l).

The drug release curves of the hydrogel coatings with different concentrations of NPs added were tested in vitro. The results showed that when the concentration of the added NPs was increased by 50 % from the initial level of 10 mg/mL, the cumulative release amount of the drug only increased by 25 %. When the concentration of NPs reached 25 mg/mL, the cumulative release amount of the drug no longer increased; instead, it was lower than that of the 15 mg/mL group. In addition, the release rate of drugs from NPs in acidic artificial urine is significantly faster than that in alkaline urine (Fig. S14). In the 10 mg/mL group, the drug release exhibited a more sustained and stable profile, with less initial burst release. We believe this phenomenon is related to the distribution of NPs in the hydrogel network. When the concentration of NPs is too high, it is difficult to achieve uniform distribution in the hydrogel network by mechanical stirring. Due to gravity and the charge interaction between particles, NPs are more likely to aggregate on the surface of the hydrogel, resulting in an increase in the initial burst release of the drug. Finally, we selected the 10 mg group for subsequent cell experiments and animal experiments.

### 2.4 Fibroblast proliferation suppression and biocompatibility

**Fig. 3:**
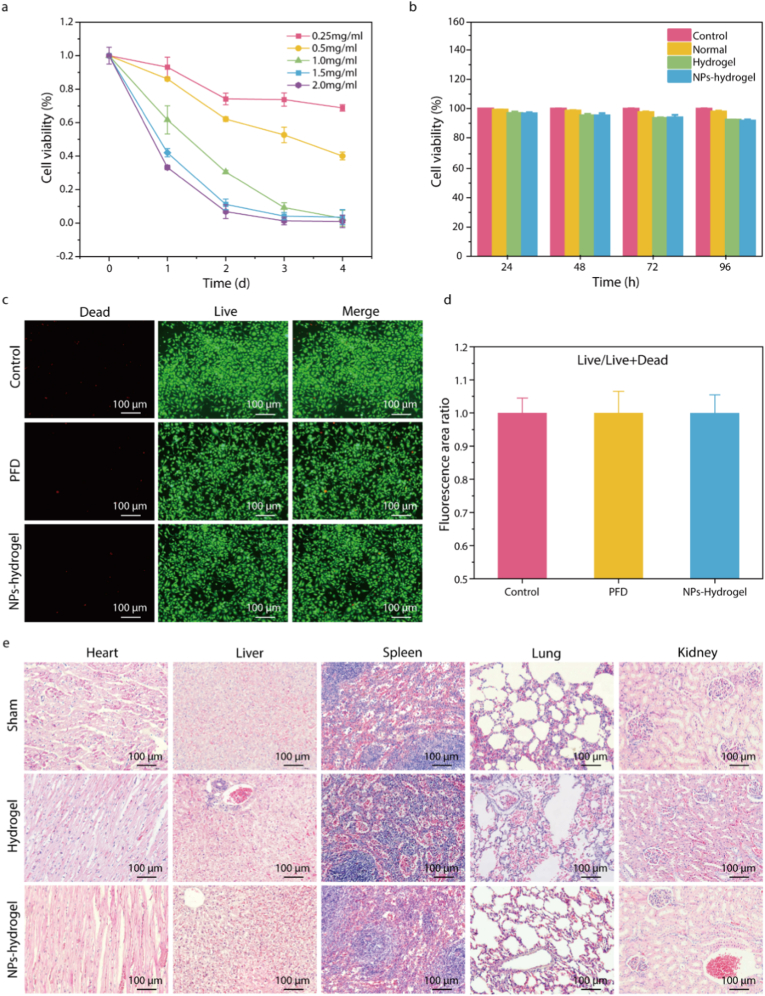
**Cell proliferation** and biocompatibility study of NPs-hydrogel coated ureteral stent. a) CCK-8 cell proliferation assay. b) CCK-8 cytotoxicity assay. c) Live/dead cell staining assay. d) Quantitative analysis of the staining assay. e) In vivo toxicity of the NPs-hydrogel coated ureteral stent. (mean ± SD, n = 3, ∗p < 0.05, ∗∗p < 0.01, ∗∗∗p < 0.001).

According to previous literature, PFD inhibits fibroblast activity and exerts anti-fibrotic effects by suppressing cell proliferation rather than inducing apoptosis or cytotoxicity [51,52]. Therefore, we examined the effect of PFD on the proliferation ability of L-929 cells. The results showed that PFD could significantly inhibit the proliferation of L-929 fibroblast cells in a dose-dependent manner (Fig. 3a). In addition, the biocompatibility of NPs-hydrogel coated stent was assessed using mouse ureteral epithelial cells, and CCK-8 and Calcein-AM/PI (live/dead) staining assays. As shown in Fig. 3b, after 1, 2, 3, and 4 days of culture, the cell viability of both the hydrogel group and the NPs-hydrogel group was slightly lower than that of the control group, but both remained above 95 %, showing no significant cytotoxicity. To further evaluate cytotoxicity, live/dead staining was performed, where red fluorescence indicated dead cells and green fluorescence indicated live cells. The results demonstrated that after a 3-day treatment with the NPs-hydrogel coated ureteral stent, only a minimal number of dead cells were observed under fluorescence microscopy (Fig. 3c and d). The above experimental results can fully demonstrate that the material does not exhibit significant cytotoxicity.

The in vivo toxicity of the NPs-hydrogel coated stent was evaluated by pathological analysis of the organs using HE staining in rabbit ureteral stricture model. After 4 weeks of stent implantation, the tissue sections of the liver, heart, spleen, lung and kidney of the rabbits were morphologically normal and showed no significant difference from the control group (Fig. 3e). These results consistently indicate that the NPs-hydrogel coated ureteral stents have good biocompatibility.

### 2.5 Assessment of anti-fibrotic, antioxidant, and anti-inflammatory effects in vitro

PFD has been proven to have anti-fibrotic, anti-inflammatory and anti-oxidative stress effects. Previous experimental studies have found that the TGFβ-1/Smad/MAPK pathway plays a significant role in ureteral fibrosis and stricture, and the general inhibition of this pathway may explain the broad anti-fibrotic effects of PFD on various fibrosis [52]. In this study, we used TGF-β and H_2_O_2_ to respectively induce L-929 cells to evaluate the in vitro effects of NPs-hydrogel coated ureteral stents.

**Fig. 4:**
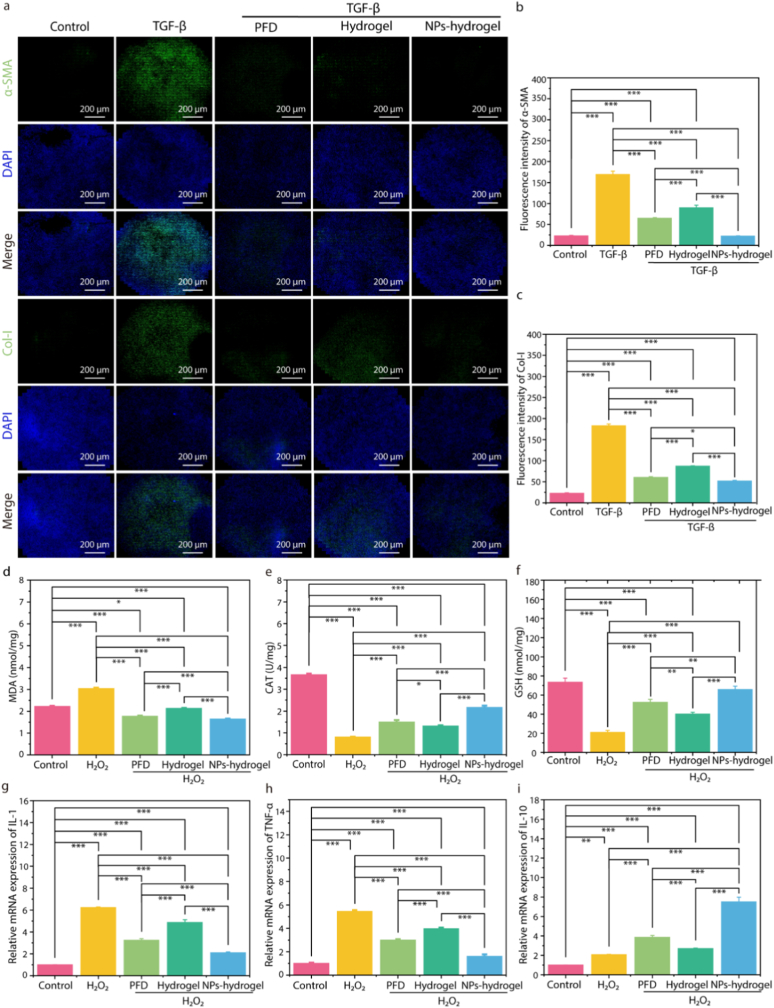
Assessment of anti-fibrotic, antioxidant, and anti-inflammatory effects in vitro. a-c) Immunofluorescence staining and quantitative analysis of α-SMA and COL-I proteins. d-f) Levels of oxidative stress-related indicators (MDA, GSH and CAT). g-i) Relative expression of inflammation activation-related genes (IL-1, TNF-α and IL-10) was examined by RT-qPCR. (mean ± SD, n = 3, ∗p < 0.05, ∗∗p < 0.01, ∗∗∗p < 0.001).

TGF-β is a pleiotropic cytokine that can stimulate the activation and proliferation of fibroblasts, significantly promote the excessive accumulation of extracellular matrix components such as α-SMA and collagen, and facilitate the formation of fibrosis [53,54]. To investigate the anti-fibrotic effect of NPs-hydrogel coated stents in vitro, the expression levels of α-SMA and Col-I were measured by IF staining. The results showed that TGF-β group exhibited high fluorescence intensity, indicating high expression levels of α-SMA and Col-I, while NPs-hydrogel group significantly inhibited the expression of α-SMA and Col-I (Fig. 4a–c). Additionally, we quantified the expression levels of fibrosis-related proteins via Western blotting. Consistent with findings from the immunofluorescence assay, under TGF-β1 stimulation, the expression of fibrosis-related proteins in the experimental group was markedly decreased. Notably, the NPs-hydrogel group exhibited the most pronounced inhibitory effect (Fig. S15).

H_2_O_2_ is a highly stable ROS that can easily diffuse into cells and is considered an important factor causing tissue oxidative stress and inflammatory responses [53,55]. We used H_2_O_2_ to induce cells to evaluate the protective effect of NPs-hydrogel coated ureteral stent on L-929 cells under oxidative stress. Malondialdehyde (MDA) is a biomarker of lipid oxidation levels in organisms. Glutathione (GSH) is a key antioxidant in cells that can protect cells from oxidative damage. Catalase (CAT) is a ubiquitous antioxidant enzyme whose main function is to remove hydrogen peroxide from the body, thereby protecting cells from the toxicity of H_2_O_2_ and is one of the key enzymes in the biological antioxidant system. The levels of MDA, CAT and GSH were measured to assess the oxidative stress levels of each group. As shown in Fig. 4d, the MDA level of L-929 cells induced by H_2_O_2_ was significantly increased and was much higher than that of the control group. After treatment with H_2_O_2_ + PFD, H_2_O_2_ + hydrogel, and H_2_O_2_ + NPs-hydrogel, the MDA levels were reduced to varying degrees. Moreover, the MDA level in the H_2_O_2_ + NPs-hydrogel group decreased the most compared with the H_2_O_2_ group. In contrast, the detection results of MDA, CAT and GSH levels showed that the NPs-hydrogel group was higher, close to the control group, and much higher than the H_2_O_2_ group (Fig. 4d–f). These data indicated that NPs-hydrogel group significantly alleviated the oxidative stress response in H_2_O_2_-induced L-929 cells. In addition, H_2_O_2_ can enhance the gene expression of inflammatory cytokines in L-929 cells, while the NPs-hydrogel group can significantly reduce the expression of inflammation-related mRNA and upregulate that of anti-inflammatory mRNA (Fig. 4g–i). Furthermore, we examined the protein expression levels associated with inflammation and oxidative stress. Western blotting results indicated that, compared with other experimental groups, the NPs-hydrogel group significantly downregulated the protein expression of IL-1β and TNF-α while upregulating that of IL-10 and Nrf2. These findings suggested that the NPs-hydrogel group can effectively mitigate intracellular fibrosis, oxidative stress, and inflammation (Fig. S15).

### 2.6 In vivo efficacy of NPs-hydrogel coated ureteral stent in a rabbit ureteral stricture model

**Fig. 5:**
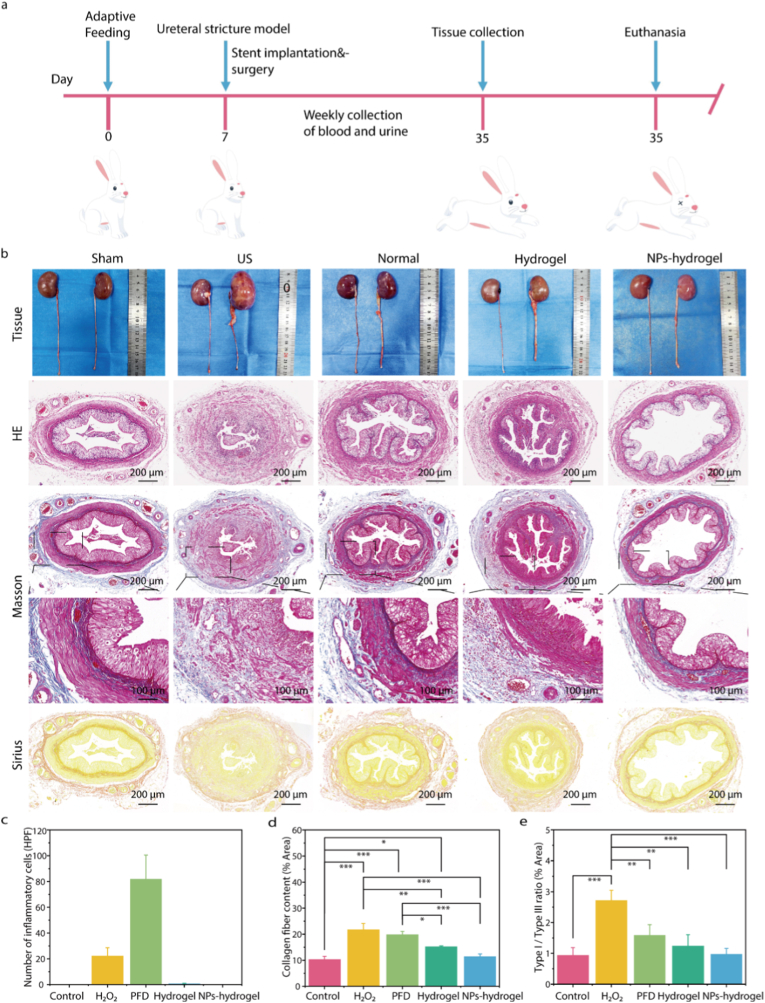
Evaluation of hydronephrosis, inflammatory infiltration and fibrosis levels in vivo. a) Schematic diagram of the animal experiment design. b) Appearance of renal and ureteral tissues, HE staining, Masson staining and Sirius Red staining. c-e) Quantitative analysis of inflammatory cell infiltration and collagen fiber content. The NPs-hydrogel coated ureteral stent demonstrated significant anti-ureteral stricture effects in vivo. (mean ± SD, n = 3, ∗p < 0.05, ∗∗p < 0.01, ∗∗∗p < 0.001).

Based on the scheme shown in Fig. 5a, we evaluated the therapeutic effect of the NPs-hydrogel coated ureteral stent on the rabbit model of US caused by electrocoagulation and mechanical injury. The concrete modeling procedure is presented in Fig. S16.

As shown in Fig. 5b, the gross appearance of the kidneys and ureters of rabbits in different groups was observed after 4 weeks post-operation. The degree of hydronephrosis and ureteral obstruction in the NPs-hydrogel coated stent group, normal stent group and hydrogel coated ureteral stent group was significantly less than that in the US group. The degree of ureteral obstruction in the NPs-hydrogel coated stent group was better than that in the other stent groups. One week after implantation, blood samples from the rabbits were collected for testing. The levels of white blood cells, neutrophils and procalcitonin in the blood of rabbits in the NPs-hydrogel coated stent group were significantly lower than those in the model group. The results showed that the NPs-hydrogel coated stent group demonstrated reliable anti-inflammatory effects in the early stage of the wound (Fig. S17). We observed the state of the hydrogel coating on the stent surface before and after the animal experiment, and found that the color of the stent turned darker yellow one month after implantation in the animal body, while there was no obvious damage to the morphology of the coating (Fig. S18).

We evaluated the morphological changes of ureteral tissues through pathological section staining. HE staining showed that the pathological features of the ureteral stricture group rabbits were severe, including obvious lumen narrowing, disordered cell arrangement, mucosal cell shedding, and significant inflammatory cell infiltration (Fig. 5b). After 4 weeks of in vivo retention, the ureteral lumen cells of the NPs-hydrogel coated stent group rabbits had normal morphology, with distinct cell arrangement layers and clear structure, and no obvious stricture. In addition, there was no obvious inflammatory response in the lumen of the NPs-hydrogel coated stent group and the hydrogel coated stent group, which was significantly better than that of the normal stent group and the US group. Traditionally, urinary tract infection is a common complication after ureteral stent placement, which is closely related to the colonization of bacteria on the surface of the ureter and the formation of biofilms [25,26]. In this work, the NPs-hydrogel coated stent and hydrogel coated stent demonstrated excellent anti-inflammatory performance, which might be related to its smooth surface that is unfavorable for bacterial colonization (Fig. 5c).

Masson and Sirius red staining were used to detect the distribution of collagen in the ureter. In model group, extensive collagen deposition was observed. In normal stent group, hydrogel coated stent group, and NPs-hydrogel coated stent group, collagen deposition was reduced to varying degrees, but the NPs-hydrogel coated stent group showed the best effect in reducing collagen deposition in the ureteral tissue (Fig. 5b–d). Type I collagen fibers serve as the basis for scar formation. The proliferation of type I and type III collagen fibers pervades the entire process of scar formation. The proportion of type I collagen fibers markedly rises, ultimately giving rise to a structure of scar tissue that is starkly dissimilar to that of normal tissue. Consequently, suppressing the excessive proliferation of type I collagen fibers can mitigate scar hyperplasia and facilitate scarless wound repair [56]. We compared the content of type I and type III collagen fibers in different groups through polarized light microscopy. The proportion was the highest in the US group and the lowest in the sham group. Among the different treatment groups, the proportion was the lowest in the NPs-hydrogel coated stent group (Fig. 5e; Fig. S19).

**Fig. 6:**
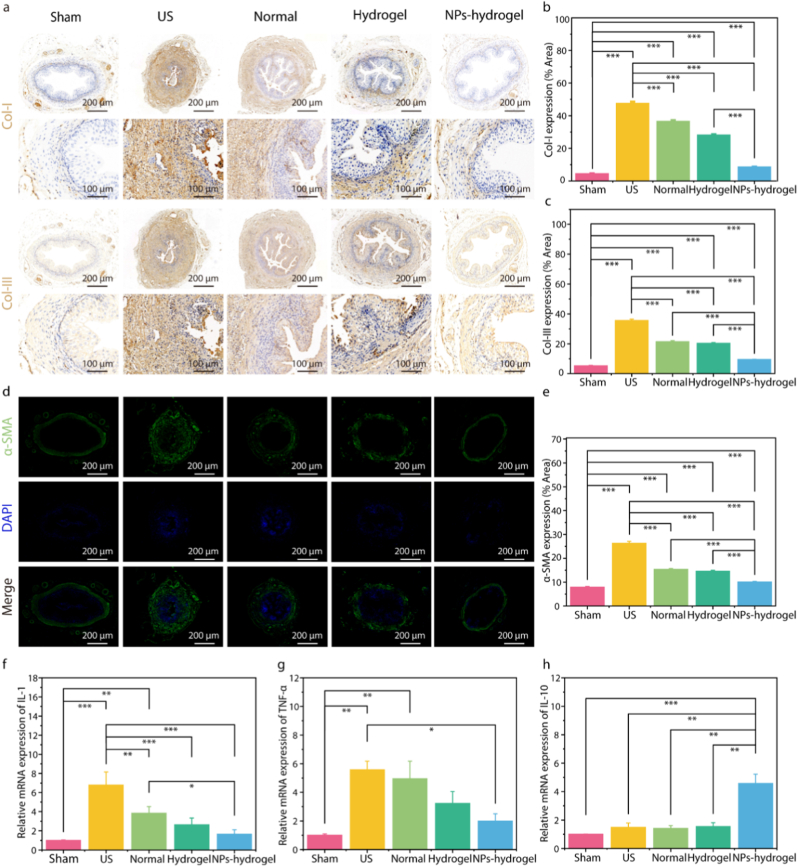
Evaluation of ureteral stricture in vivo. a-c) Immunohistochemical staining and quantitative analysis of Col-I and Col-III. d-e) Immunofluorescence staining and quantitative analysis of α-SMA protein. f-h) Relative expression of inflammation-related genes (IL-1, TNF-α and IL-10) was examined by RT-qPCR. The ureters of rabbits in the US group showed typical fibrotic and inflammatory pathological changes, and the NPs-hydrogel coated ureteral stent group exhibited excellent anti-fibrotic and anti-inflammatory effects. (mean ± SD, n = 3, ∗p < 0.05, ∗∗p < 0.01, ∗∗∗p < 0.001).

To further confirm this, IHC and IF staining were used to detect the expression of three important markers of fibrosis, α-SMA, Col-I, and Col-III (Fig. 6a–e). Consistent with the above results, the positive areas of α-SMA, Col-I, and Col-III in the ureteral tissue of the model group were significantly increased. The normal stent group, hydrogel coated stent group, and NPs-hydrogel coated stent group could all reduce the expression of α-SMA, Col-I, and Col-III, among which the NPs-hydrogel coated stent group had the most significant effect, approaching that of the Sham group. The qRT-PCR was used to detect the expression of inflammatory cytokines. Both the hydrogel stent group and the NPs-hydrogel coating stent group could reduce the expression of IL-1β, TNF-α, and increase the expression of IL-10, with the NPs-hydrogel coated stent group showing the best effect (Fig. 6f–h). These results indicated that the NPs-hydrogel coated stent can reverse the pathological changes related to ureteral stricture caused by iatrogenic injury. However, in the present study, the rabbit ureteral stricture model was only a short-term 4-week trial, which fails to validate the long-term efficacy of the stent. In further studies, we will extend the experimental duration to explore the long-term preventive and therapeutic effects of the novel stent on ureteral stricture.

## 3 Conclusions

In summary, we developed a NPs-hydrogel coated ureteral stent for the prevention of iatrogenic US. The PAM hydrogel coating demonstrated high mechanical properties, biocompatibility, and antifouling performance. The PFD-PLGA NPs decorated with acryloyl functional groups were firmly anchored within the hydrogel network, enabling sustained drug release. In vitro cell experiments, NPs-hydrogel coated stent inhibited fibroblast proliferation and extracellular matrix deposition, significantly eliminated ROS, and suppressed the expression of inflammatory cytokines. In the rabbit US model, the NPs-hydrogel coated ureteral stent demonstrated a powerful anti-stricture effect by inhibiting fibrosis, alleviating oxidative stress and reducing the expression of inflammatory cytokines. Overall, our research has demonstrated the preventive and therapeutic effects of NPs-hydrogel coated ureteral stents on iatrogenic US and developed a drug sustained-release delivery platform with great potential for clinical application.

## 4 Experimental section

### 4.1 Synthesis and characterization of functionalized PLGA (fPLGA)

To graft double bonds at the end of PLGA-PEG-NHS, 0.1 mol of PLGA-PEG-NHS and an excess of AEM (1mol) were dissolved in dimethyl sulfoxide (2 mL). The pH of the solution was adjusted to weakly alkaline (pH 8.5–9.0) with potassium hydroxide, and the reaction was carried out in the dark at room temperature for 12 h. Then, an excess of deionized water was added to precipitate the product, which was further collected and purified by centrifugation and freeze-drying. The successful chemical modification of fPLGA was confirmed by FTIR spectroscopy and NMR-H.

### 4.2 Synthesis and characterization of PFD-fPLGA NPs

Double emulsion solvent evaporation technique was chosen to prepare PFD-fPLGA NPs. Briefly, a solution of PFD in PBS (0.5 mL) was added to DCM solution including fPLGA (20 mg). The mixed solution was emulsified in an ice-bath using an Ultrasonic Homogenizer (SCIENTZ, 950E) for 5 min. The W/O emulsion was emulsified into a PVA solution (10 ml, 0.5 % PVA) in an ice-bath sonicating for 10 min. Next, DCM was completely evaporated after magnetic stirring for 8 h at room temperature. Large particles and precipitated drugs were eliminated through centrifugation at 5000 rpm for 10 min, while NPs were isolated from the supernatant by further centrifugation at 20000 rpm at 4 °C for 30 min. The synthesized NPs were pre-frozen in a −80 °C refrigerator containing 5 % mannitol as a cryoprotectant for 2 h, and then processed in a vacuum freeze dryer for 24 h. The dried NPs were subsequently stored at 4 °C.

The encapsulation efficiency and drug loading of NPs were characterized by an UltiMate3000 ultra-performance liquid chromatograph (HPLC) at a wavelength of 312 nm. The morphology and particle size of NPs were observed by transmission electron microscopy (TEM, JEM-2800) and atomic force microscopy (AFM, Dimension Icon). Fourier transform infrared spectroscopy (FTIR, Nicolet iS50) was used for spectral analysis. The particle size and potential distribution of NPs were detected by a nanoparticle size and zeta potential analyzer (Malvern Zetasizer, Zano ZS).

### 4.3 Preparation and characterization of NPs-hydrogel coated ureteral stent

Ureteral stent was cleaned in deionized water and ethyl alcohol for 3 times by ultrasonic cleaner. Immerse the ureteral stent in a 10 wt% solution of benzophenone in ethanol for 10 min. 20 wt% acrylamide, 1 wt% of Irgacure 2959 and appropriate amounts of NPs were dissolved in deionized water, and then fully mixed using a magnetic stirrer for 10 min. Further, the ureteral stent is immersed in the mixed solution and illuminated under ultraviolet light for 35 min to obtain the final product. Finally, rinsing the stent with deionized water for 1 h to remove unreacted reagents and components.

The morphology and structure of the freeze-dried NPs-hydrogel coated ureteral stent were observed using scanning electron microscopy (SEM; JSM 7800F). The surface morphology of NPs-hydrogel coated ureteral stent in different states and time points was photographed by camera. The ureteral stents were stained with red dye and photographed by microscope. Subsequently, NPs-hydrogel coatings were prepared on the polyurethane substrate (2 cm × 2 cm) to further evaluate the mechanical properties. The elastic modulus of samples was obtained by nanoindentation mode of AFM with 50 nm indentation depth. Friction coefficients of samples were measured by a rotary rheometer in normal force control mode using a 10 mm steel parallel plate clamp. Then, each sample was loaded into a rheometer and atmospheric pressures (3 kPa) was applied to the sample immersed in a deionized water bath at a steady shear rate of 0.5–1 s. Additionally, water contact angles of polyurethane films with and without hydrogel coatings were evaluated using a contact Angle meter. The degradation rate and stability of the NPs-hydrogel coating were evaluated by recording the mass loss and swelling ratio changes of the hydrogel coating at different time points in artificial urine medium. The samples were co-cultured with Escherichia coli (E. coli). After staining, a laser confocal microscope was used to compare the number of bacteria adhering to the surface, thereby evaluating the antifouling performance.

### 4.4 In vitro drug release study

The in vitro drug release behavior of PFD was determined using the dynamic dialysis method. The stent was cut into 1 cm segments, which were then immersed separately in 4 mL of PBS (pH 7.4) and artificial urine (pH 4.7 and 7.4), followed by the addition of 0.02 wt% sodium azide. Subsequently, the mixture was transferred into a dialysis bag (molecular weight cut-off: 3.5 kDa), and the dialysis bag was moved to a glass beaker containing 100 mL of the corresponding solvent. The glass beaker was continuously shaken at 100 rpm in an incubator at 37 °C. At specified time points, 4 mL of the solution outside the dialysis bag was replaced with an equal volume of fresh solvent. The sample concentration was determined by measuring the ultraviolet–visible (UV–VIS) absorbance at 312 nm.

### 4.5 Biocompatibility assessment

The cytocompatibility of the stent was evaluated by Cell Counting Kit-8 (CCK-8) and live-dead cell staining. After sterilization, the ureteral stent was immersed in liquid culture medium for 24 h. The extract was obtained after centrifugation and filtration. Mouse ureteral epithelial cells were seeded into 96-well plates at a density of 100 μL/well. After incubation for 24, 48, 72, and 96 h, CCK-8 reagent was added to stain the cells. Then, the cells were incubated in the dark for 2 h, and the OD value at 450 nm was measured using a microplate reader. Additionally, the cells were seeded onto 24-well tissue culture plates at a density of 1 × 10^6^ cells/well in the same manner, and incubated with a mixed solution of Hoechst 33342 and PI. The cell viability was directly evaluated by fluorescence microscopy, and the microscopic images were quantitatively analyzed using ImageJ software.

*Cell Proliferation Test:* L-929 cells were cultured in 96-well plates and divided into control group and drug group. The drug group was simply added with medium containing pirfenidone (0.25, 0.5, 1.0, 1.5, 2.0 mg/mL). The incubation time gradient was set at 24, 48, 72, and 96 h, and after the medium was removed, 10 % CCK-8 was added. The solution was incubated for 2 h and the 450 nm absorption wavelength was detected using a microplate reader.

*In Vivo Toxicity Study:* Heart, liver, spleen, lung and kidney specimens from different groups of rabbits were collected. The changes in different organs were observed through HE staining method to evaluate the in vivo toxicity of the stent.

### 4.6 Assessment of NPs-hydrogel coated ureteral stent effect in vitro

The anti-fibrosis, anti-inflammatory and anti-oxidative stress effects of PFD were evaluated in vitro. First, the anti-fibrosis effect of PFD was assessed in TGF-β-induced L-929 cells. The expression levels of α-SMA, Col-I and Col-III were detected by immunofluorescence (IF) staining. The cells were seeded in 6-well plates, and divided into five groups. Cells were fixed with 4 % paraformaldehyde for 15 min, blocked with PBS containing 5 % FBS and 0.3 % Triton™X-100 for 1 h, and incubated overnight at 4 °C with primary antibodies α-SMA, Col-I and Col-III. Then, they were incubated with fluorescent dye-conjugated secondary antibodies for 1 h at room temperature in the dark. The cell nuclei were stained with DAPI for 5 min. Graphic images were obtained through a fluorescence microscope. Second, the antioxidant effect of PFD was assessed in H_2_O_2_-induced L-929 cells. Cells were seeded in 6-well plates and divided into five groups based on the different interventions. Cells suspended in the extraction buffer were lysed using ultrasound. After centrifugation at 10000×*g* for 15 min, the supernatant was used for catalase (CAT) activity assay reagent, glutathione (GSH) assay kit and malondialdehyde (MDA) content assay kit to assess oxidative stress levels. The anti-inflammatory effect of PFD was assessed by RT-qPCR. The expression levels of TNF-α, IL-1β, IL-6, and IL-10 were detected by IF staining.

### 4.7 Animal experimentation

All animal experiments were conducted in accordance with the guidelines approved by the Animal Protection and Use Committee of Tianjin Jinke Biotechnology Co., Ltd. (IACUC Number: GENINK-20240011). Twenty-five male New Zealand rabbits aged 20 weeks were selected as the experimental model. The rabbits were randomly sorted into five groups: US, Sham, US + normal ureteral stent, US + hydrogel stent, US + NPs-hydrogel stent. All rabbits were randomly numbered and fasted for 2 h. The prepared ureteral stent was sterilized with low temperature ethylene oxide. All rabbits were administered intramuscular injections of Zoletil 50 (0.5 mL/kg) and Sumianxin II (0.3 mL/kg). Following the induction of general anesthesia, the rabbits were positioned supine on the operating table, where a median abdominal incision was made. Upon entering the abdominal cavity, intestinal contents were retracted to facilitate separation of adipose tissue surrounding the ureter, allowing for approximately 3 cm of ureter to be freed. Using microsurgical scissors, an incision was made in the ureteral lumen; electrodes were then inserted to electrocauterize a full thickness segment of approximately 1 cm for 2 s. The intestine was repositioned before closing the abdominal cavity. Postoperative care included administration of antibiotics and analgesics. Blood and urine samples were collected at 1 and 2 weeks after surgery. After 4 weeks, ureteral tissues and organs were harvested for histological analysis.

HE staining was employed to observe the stricture of the ureteral lumen and assess the infiltration of inflammatory cells within the ureter. Masson's trichrome staining and Sirius red staining were used to observe the fibrosis of the ureter, and the expressions of α-SMA, Col-I, and Col-III at the injury site were detected. Furthermore, the expression of the above-mentioned proteins was analyzed through immunohistochemistry (IHC) and IF staining. Quantitative analysis of fluorescence images was performed using Image J software to measure the average fluorescence intensity, which was determined by dividing the total fluorescence intensity measured in a specific area by the area.

### 4.8 Biochemical indicators

Serum levels of white blood cell (WBC), neutrophile granulocyte (NEU), procalcitonin (PCT), hemoglobin (HB), red blood cell (RBC), creatinine (CRE), alanine aminotransferase (ALT), aspartate aminotransferase (AST), γ-glutamyl transpeptidase (GGT) and blood glucose were measured using an Auto hematology analyzer.

### 4.9 qRT-PCR measurement

The expression levels of TNF-α, IL-1β, and IL-10 in the ureter tissue were examined by RT-PCR. Total RNA was extracted from the tissues of each group of rabbits using TRIzol. Reverse transcription reactions were carried out using the Takara reverse transcription kit with the RNA of each group as the template to synthesize the first strand of cDNA. qRT-PCR was conducted in a reaction system (20 μL) using the SYBR Premix Ex TaqTM II kit. Primers for all genes were listed in Table S2. The expression level of the GAPDH gene was used as an internal reference for each sample, and the relative mRNA expression level of the target gene in each sample was calculated by the relative quantification (2^−ΔΔCT^) method.

### 4.10 Statistical analysis

All experiments were repeated at least 3 times for each sample. Statistical analysis was performed using Origin 2025 software, including one-way analysis of variance and Tukey's post-hoc test. Data were presented as mean ± standard deviation. The statistical significance threshold was set at P < 0.05, and asterisks were used to indicate statistical significance (∗, p < 0.05; ∗∗, p < 0.001; ∗∗∗, p < 0.001).

## CRediT authorship contribution statement

**Dawei Tian:** Writing – original draft, Visualization, Validation, Project administration, Methodology, Formal analysis, Data curation, Conceptualization. **Jian Wang:** Writing – review & editing, Writing – original draft, Visualization, Validation, Supervision, Methodology, Funding acquisition, Formal analysis, Data curation. **Guoqiang Han:** Validation, Investigation, Formal analysis, Data curation. **Weiwei Wang:** Methodology, Formal analysis, Data curation. **Lei Cui:** Methodology, Formal analysis. **Chenning Li:** Formal analysis, Data curation. **Wei Wang:** Formal analysis, Data curation. **Zhenhua Yang:** Conceptualization. **Diansheng Zhou:** Supervision. **Xiepeng Zuo:** Data curation. **Zesheng An:** Supervision. **Tianxiao Xu:** Supervision. **Jian Zhu:** Writing – review & editing, Supervision, Methodology, Funding acquisition, Conceptualization.

## Declaration of competing interest

The authors declare that they have no known competing financial interests or personal relationships that could have appeared to influence the work reported in this paper.

## Data Availability

Data will be made available on request.
