# Supplementary material for: Sustained drug-releasing hydrogel coatings for ureteral stents to prevent iatrogenic injury-induced ureteral stricture
Source: Mater Today Bio. 2025 Oct 28;35:102481. doi: 10.1016/j.mtbio.2025.102481 (PMC12642134; doi:10.1016/j.mtbio.2025.102481)
Supplement: Multimedia component 1 [file mmc1.docx]

Supporting Information

**Materials**

Ureteral stents were obtained from Cook Medical (USI-626-CE, 6.0Fr) and Oukai Medical Instrument Co., Ltd. (Customized, 2.4Fr). All chemicals were used as received without further purification. N-Hydroxysuccinimide poly (ethylene glycol)-block-poly(lactide-co-glycolide) (PEG average Mn 300-400, PLGA average Mn 10000, lactide: glycolide 50:50) was custom-synthesized by Yusi Pharmaceutical Co., Ltd (Chongqing China). Poly (vinyl alcohol) (PVA; MW 30,000-70,000; 88% hydrolyzed), pirfenidone (PFD，≥97%), 2-aminoethyl methacrylate hydrochloride (AEM), phosphate buffer saline (PBS, pH7.4), were obtained from Sigma Aldrich (USA). dichloromethane (DCM), ethanol absolute, hydrogen peroxide (30%), acrylamide, Irgacure-2959, Benzophenone (BP) were obtained from Macklin (China). Artificial urine was purchased from LEAGENE (China). Dulbecco’s Modified Eagle Medium (DMEM), and fetal bovine serum (FBS, C0235) were obtained from Gibco (USA). Cell counting kit-8 (CCK-8), Lipid Peroxidation MDA Assay Kit, Catalase Assay Kit and GSH Assay Kit were acquired from Beyotime (China). Collagen I (1:1000, 66761-1-Ig), Collagen III (1:1000, 22734-1-AP), α-SMA (1:1000, 67735-1-Ig), IL-1 (1:500, 26048-1-AP), TNF-α (1:500, 17590-1-AP) and GAPDH (1:2000, 60004-1-Ig) were purchased from Proteintech Group, Inc (Wuhan, China). IL-10 (1:500, DF6894) and Nrf2 (1:500, AF0639) were purchased from Affinity Biosciences. Horseradish Peroxidase (HRP) conjugated affiniPure IgG (1:1000, BA1051) and blocking serum were purchased from Boster Biological Technology Co., Ltd. (Wuhan, China). TGF-β1 (PCK099, 10 ng/mL) was purchased from Procell Life Science & Technology Co., Ltd (Wuhan, China). The blood samples were analyzed using an automatic blood cell analyzer (Mindray Company, BC-5100). Unless otherwise indicated, all antibody and detection assay kit were purchased from Proteintech group. Water used for all experiments was purified using a Milli-Q filtration system.

**Cell lines and animals**

NCTC clone 929 cells (L-929, CL-0137) and mouse ureteral epithelial cells (CP-M071) were purchased from Pricella Biotechnology Co., Ltd. (Wuhan, China). Adult male New Zealand rabbits were purchased from Jinke Biotechnology Co., Ltd. (Tianjin, China). All rabbits were placed in a constant temperature and humidity room, with artificial light and dark cycles for 12 h, and were allowed free access to food and water.

**Table S1.** **Characteristics of nanoparticles with varying compositions**

| **Code** | **fPLGA:PFD ratio** | **MPS (nm)^a^** | **PI^a^** | **ZP (mV)^a^** | **EE (%) ^a^** | **DL (%) ^a^** |
| --- | --- | --- | --- | --- | --- | --- |
| 1 | 1:1 | 210.6±5.4 | 0.223±0.042 | -18.80±0.17 | 49.05±1.32 | 9.55± 0.16 |
| 2 | 2:1 | 248.2±5.1 | 0.299±0.027 | -17.80±0.25 | 57.44±1.66 | 10.02±0.34 |
| 3 | 3:1 | 189.3±2.5 | 0.141±0.016 | -15.20±0.75 | 66.04±2.04 | 12.41±1.08 |
| 4 | 5:1 | 210.3±1.9 | 0.204±0.020 | -15.20±0.50 | 72.04±2.92 | 11.19±1.36 |

Data are shown as mean ± standard deviation

EE entrapment efficiency, MPS mean particle size, ZP zeta potential, PI polydispersity index, DL drug loading

Determination of EE and DL: Firstly, collect the supernatant during the centrifugation of the NPs, measure the concentration of free drug in the supernatant, and the amount of drug encapsulated in the NPs is the total drug amount minus the free drug amount. Subsequently, precisely weigh 5mg of PFD-fPLGA NPs, fully dissolve them in 5ml of methanol, sonicate for 20 min, filter through a 0.22μm microporous membrane, and determine the content of PFD in the NPs by HPLC.

Chromatographic column: C18(250mm × 4.6mm, 5μm); Mobile phase: methanol - 0.5% acetic acid aqueous solution (volume ratio 4:1), column temperature 35 ℃, flow rate 1.0 mL/min, detection wavelength 312 nm, injection volume 10 μL. Further, based on previous research reports, the standard curve of PFD was plotted.

The calculation formula is as follows:

$\mathrm{EE}\left( \% \right)=\frac{m1}{\mathrm{mo}}\times100\%$ （1）

$\mathrm{DL}\left( \% \right)=\frac{m1}{m 2}\times100\%$ （2）

In the above equation: m1 represents the actual mass of PFD loaded; m0 represents the theoretical mass of PFD input; m2 represents the mass of PFD-fPLGA NPs.

^a^ All the tests were carried out in triplicate

**Table S2.** **The forward and reverse primer sequences of different genes for RT-qPCR performance**

| **Gene** | **Forward primer** | **Reverse primer** |
| --- | --- | --- |
| Mouse IL-6 | GCACTGGCGGAAGTCAATCTG | TGATTCTCAGCAGGCAGGTCTC |
| Mouse IL-10 | CGTGCTATGTTGCCTGGTCTTC | GCCGCCTGGAAAGTGAATGC |
| Mouse TNF-α | ACGTAGTAGCAAACCCGCAA | TTCTCCAACTGGAAGACGCC |
| Mouse IL-1β | GACCTGTTCTTTGAGGCCGA | TTCTCCAGAGCCACAACGAC |
| Mouse α- SMA | AGGTCGGAGTGAACGGATTTG | GCCGTGGGTGGAATCATACT |
| Mouse TGF-β1 | CATTGCTGGTCCAGTCTGCTTCG | TGGTGAATGACAGTGCGGTTATGG |
| Mouse Col-I | GACAGGCGAACAAGGTGACAGAG | CAGGAGAACCAGGAGAACCAGGAG |
| Mouse Col-III | ACGAGGTGACAAAGGTGAAACTGG | AGAACCTGGAGGACCTGGATTGC |
| Mouse GAPDH | AGGTCGGAGTGAACGGATTTG | GCCGTGGGTGGAATCATACT |
| Rabbit IL-1β | TGTCAGTCGTTGTGGCTCTG | AGTCATCCCAGGTGTTGCAG |
| Rabbit TNF-α | TTCTCTTTCCTGCTCGTGGC | GGAGGTTGTTTGGGGACTCT |
| Rabbit IL-10 | TACCTGAAGGACGTGATGCC | GAAGATGTCAAACTCACTCATGG |
| Rabbit GAPDH | GCAAAGTGGATGTTGTCGCC | TGATGACCAGCTTCCCGTTC |

**
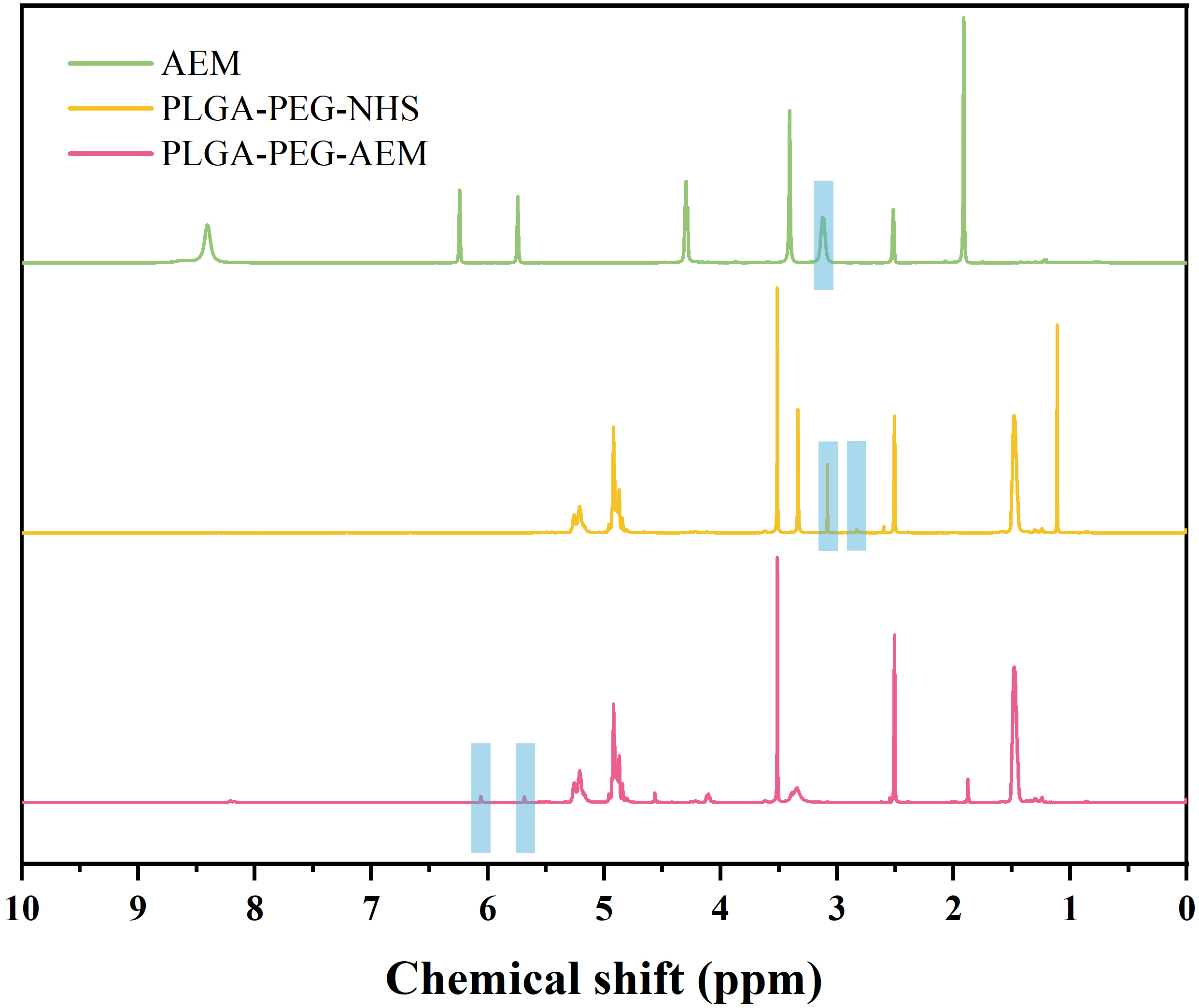
**

**Figure S1.** **1H NMR spectra of PLGA-PEG-NHS, AEM and PLGA-PEG-AEM (fPLGA).** In the proton NMR spectrum of fPLGA, the characteristic double bond peaks of AEM can be observed at 5.68 ppm and 6.05 ppm, with a chemical shift to lower field compared to the original peaks. Due to the formation of the amide bond, the methylene peak adjacent to the amino group in AEM shifts to 3.51 ppm and merges with the PEG peak. Additionally, the characteristic peak of the NHS group at 2.8 - 3.3 ppm disappears. The changes in the peaks indicate the successful synthesis of fPLGA.


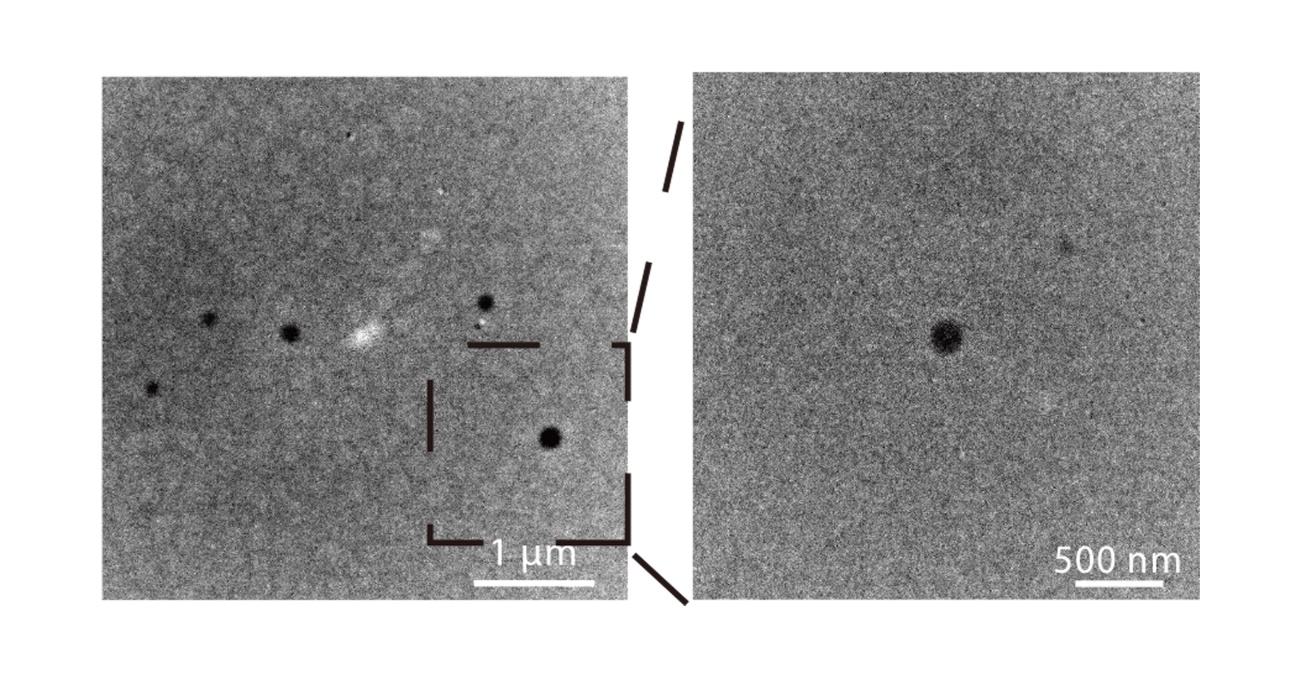


**Figure S2.** **TEM images of PFD-fPLGA NPs.**


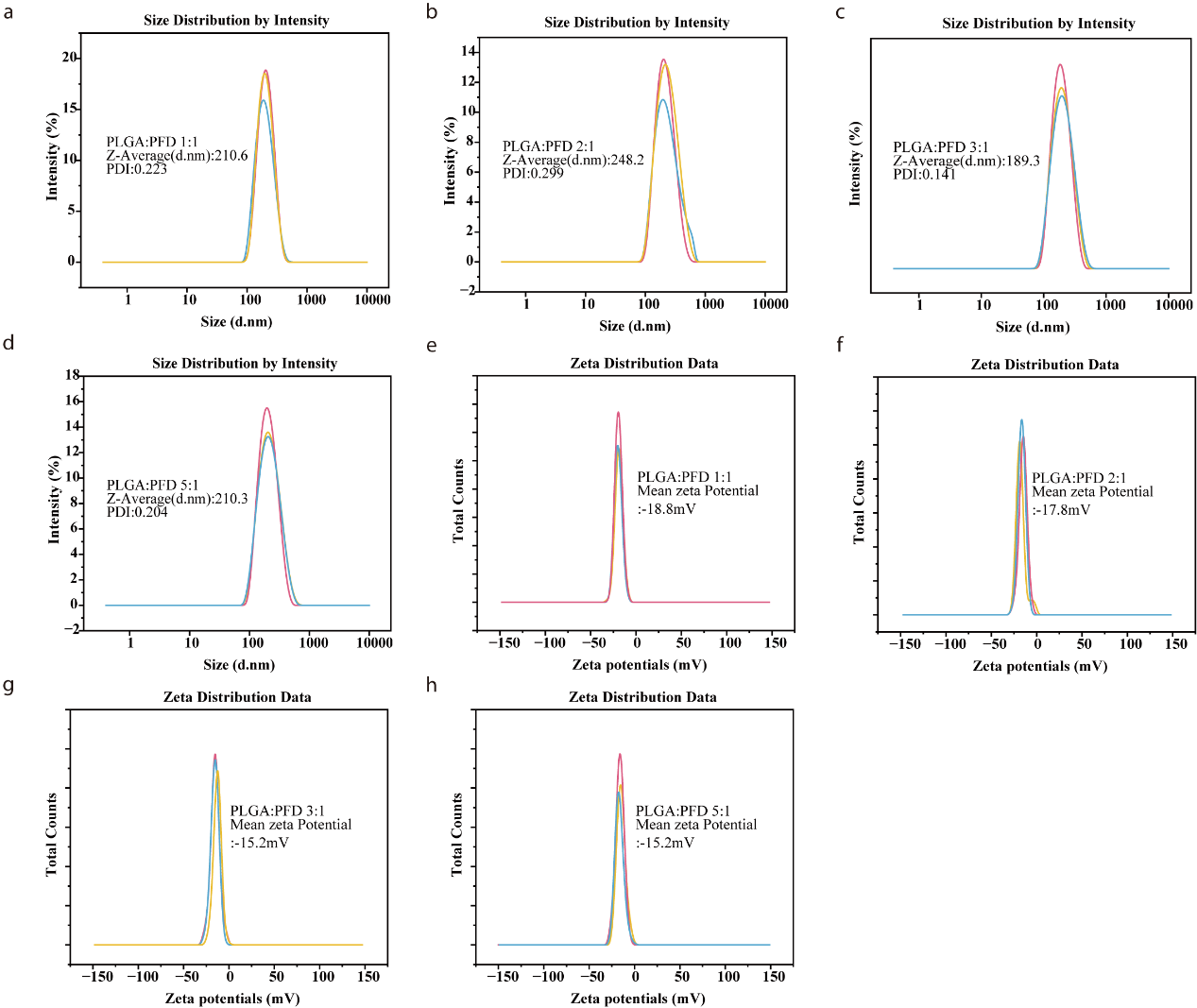


**Figure S3.** **The particle size and potential of NPs with different components.** (a-d) Size; (e-h) Zeta potential.

**
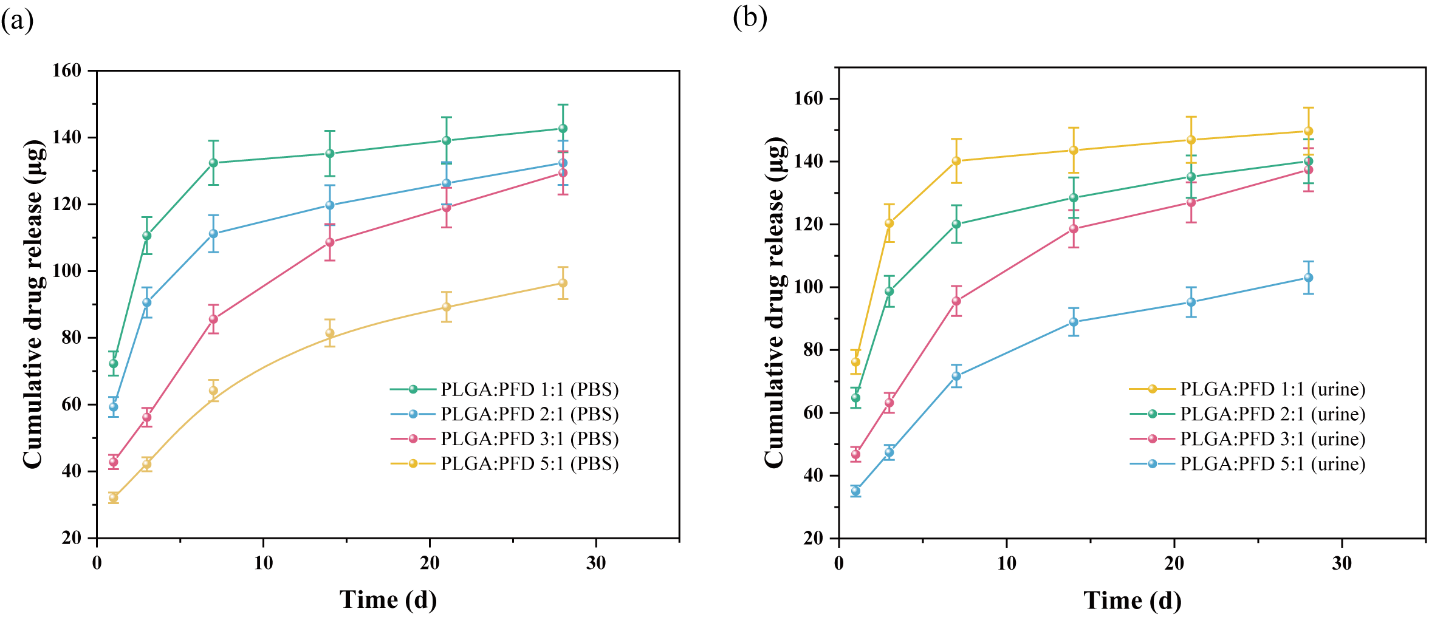
**

**Figure S4.** **In vitro drug release curves of nanoparticles of different proportions.** (a) In PBS solution environment. (b) In artificial urine environment.


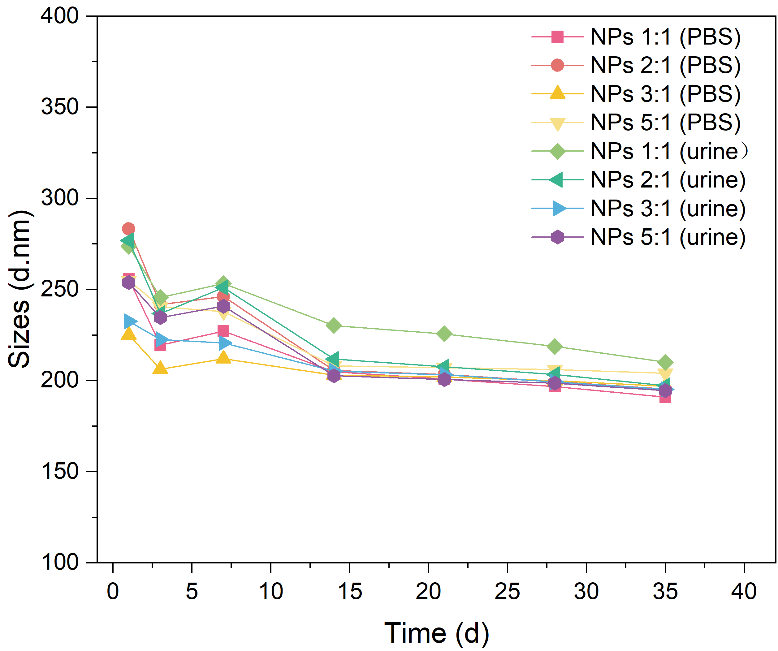


**Figure S5.** **Stability experiments of PFD-fPLGA NPs with different fPLGA and PFD ratios in PBS and artificial urine.**

**
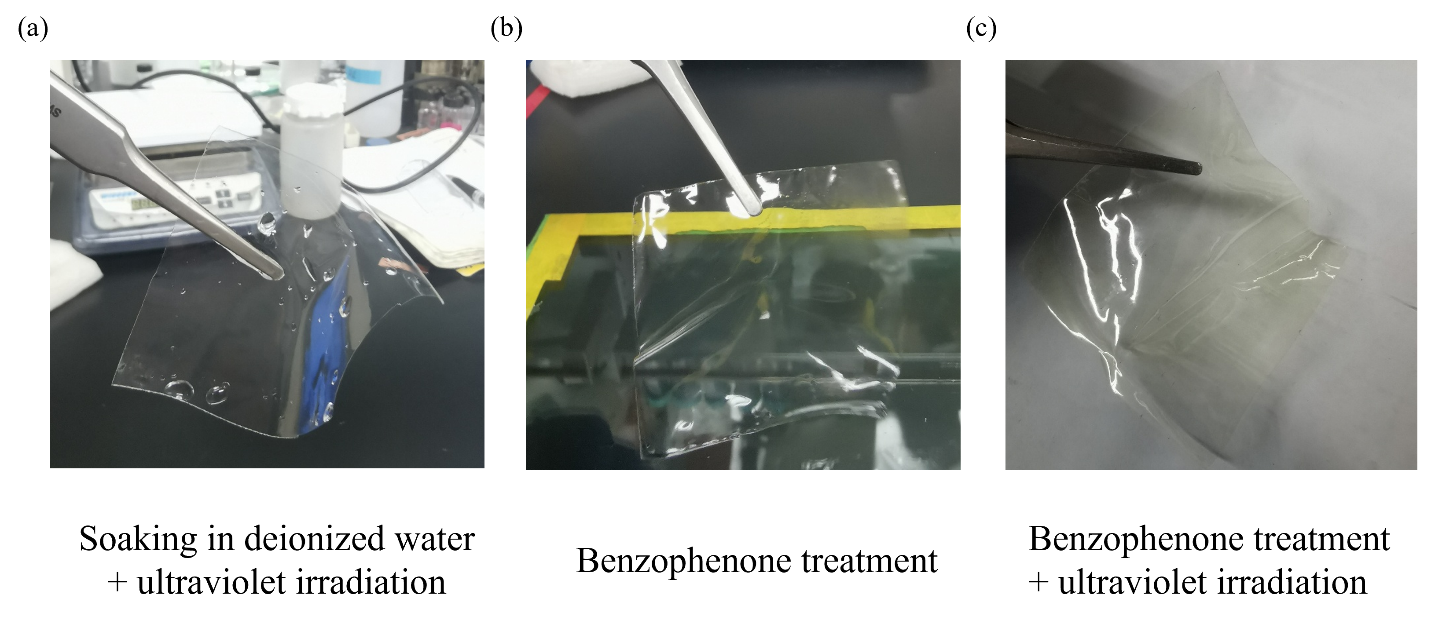
**

**Figure S6.** **Camera photo of PU film after different treatments** (a) Soaking in deionized water + ultraviolet irradiation. (b) Benzophenone treatment. (c) Benzophenone treatment + ultraviolet irradiation.

**
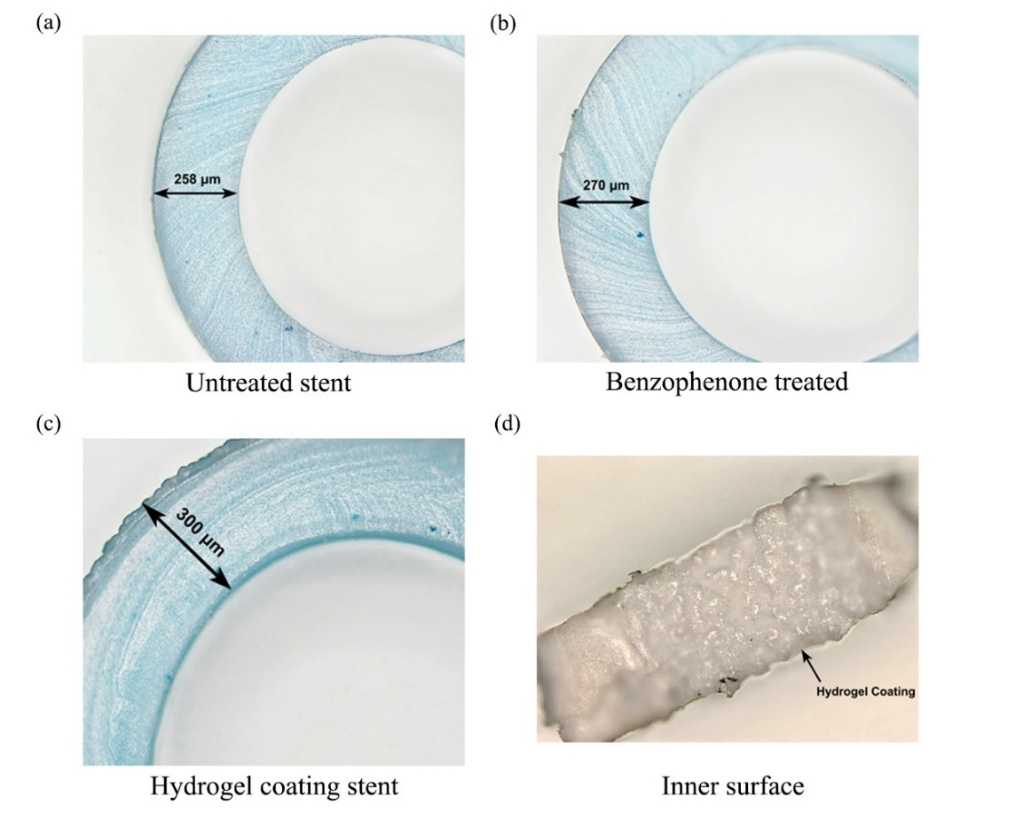
**

**Figure S7.** **Microscope images of the diameter variation in diameter and hydrogel coating on the inner surface of ureteral stent at different stages.** (a) Untreated stent; (b) Benzophenone treated; (c) NPs-hydrogel coated stent; (d) The inner surface of the ureteral stent also has hydrogel coating.


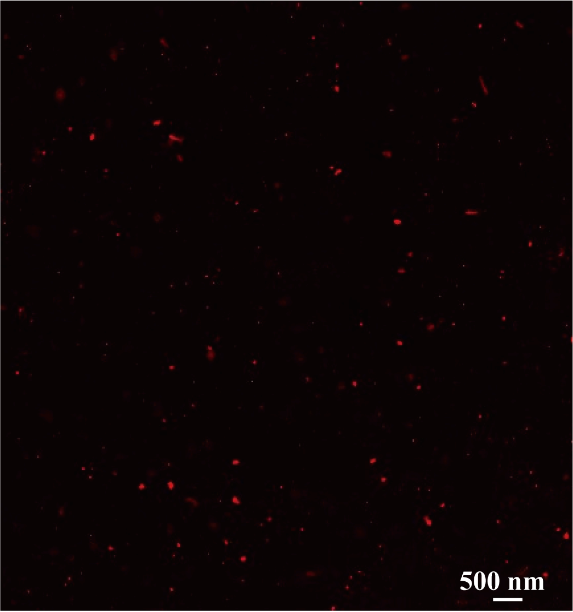


**Figure S8.** **Laser confocal microscopy images of the distribution of Nile Red-labeled PFD-PLGA NPs in PAM hydrogels.** The spatial distribution of Nile Red-labeled PFD-PLGA NPs confirms that the NPs were successfully and uniformly loaded into the hydrogel coating.


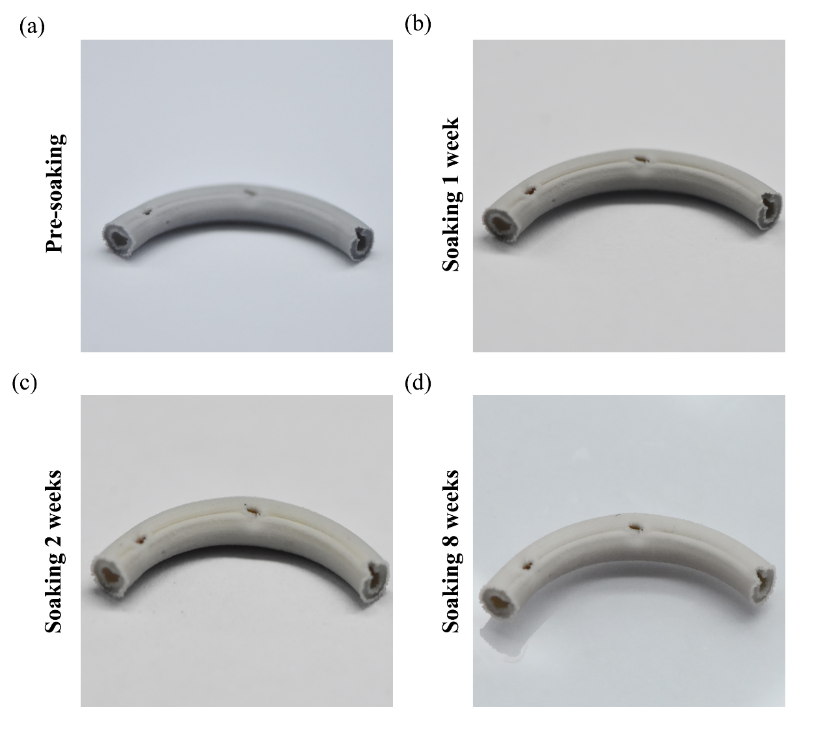


**Figure S9.** **Camera photo of ureteral stent immersed in artificial urine environment.** The NPs-hydrogel coated stent was taken out and photographed at different time points, and the changes of surface morphology were compared.


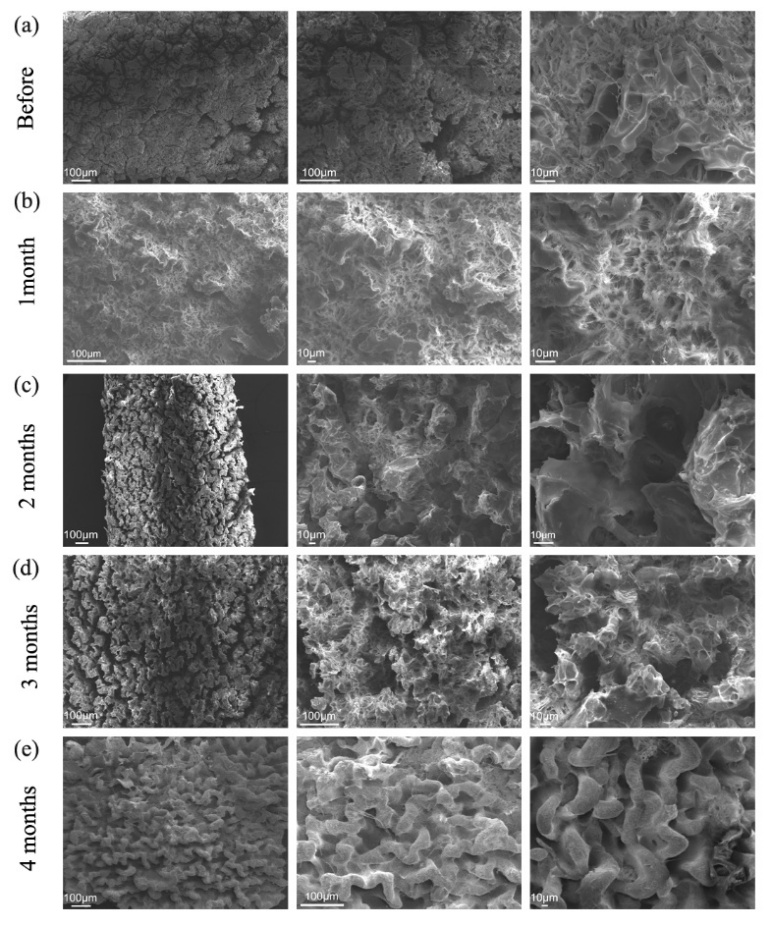


**Figure S10.** **SEM images of stent immersed in artificial urine environment for different time points.** (a) Before. (b) Soaking 1 months. (c) Soaking 2 months. (d) Soaking 4 months. (e) Soaking 6 months.


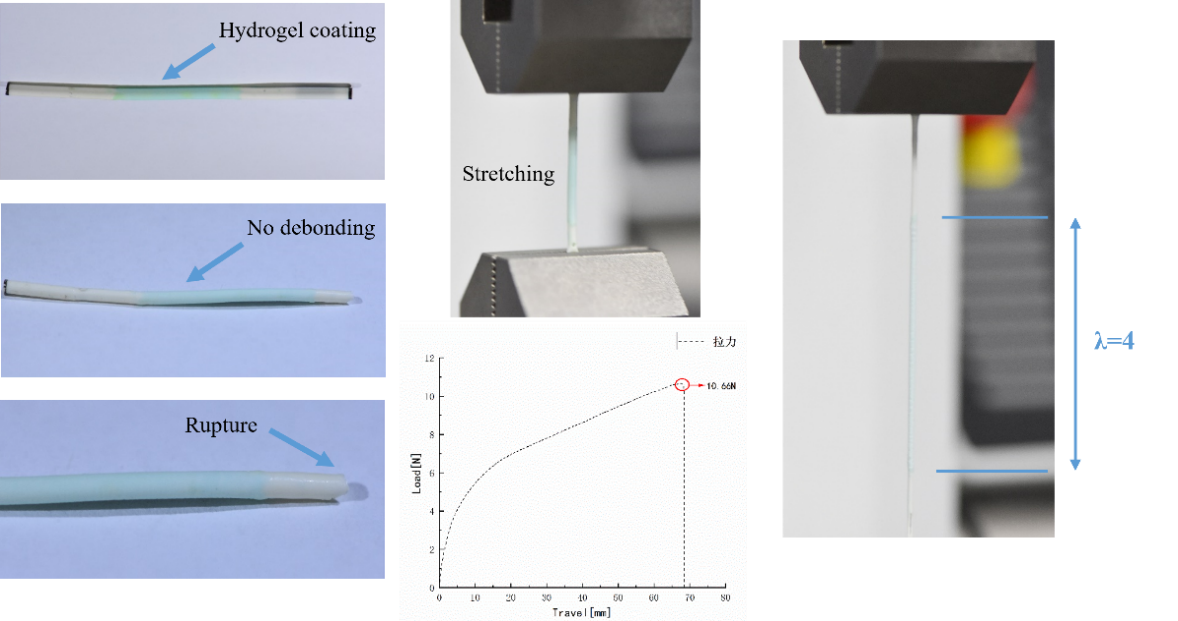


**Figure S11.** **Tensile test of the hydrogel-elastomer composite material.** The hydrogel coating can withstand large deformations (with a stretch ratio exceeding 4 times) without debonding. Even after the PU stent fractures, the robust hydrogel-elastomer bond remains intact.


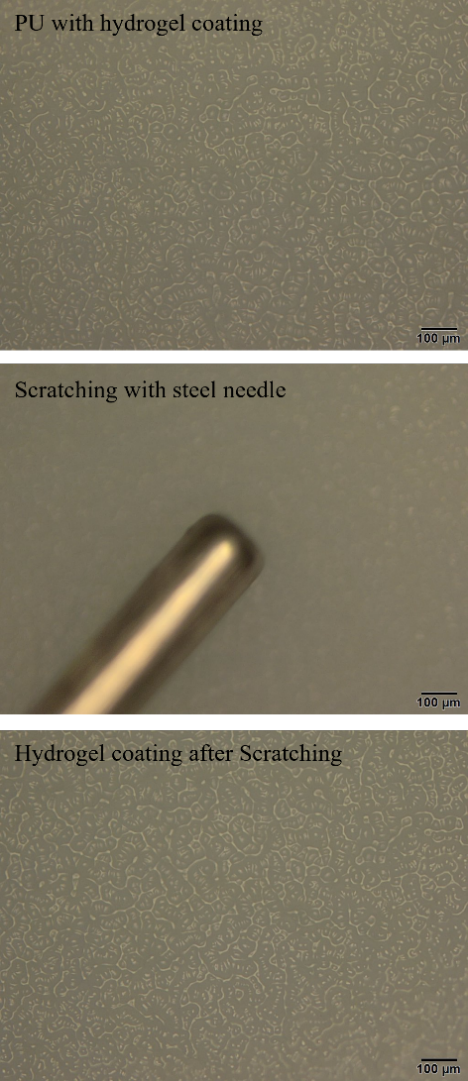


**Figure S12.** **Scratch tests were performed on the hydrogel coating using a steel needle.** Microscopic images of the scratch tests conducted with a stainless steel needle are provided to demonstrate the mechanical strength of the hydrogel coating.


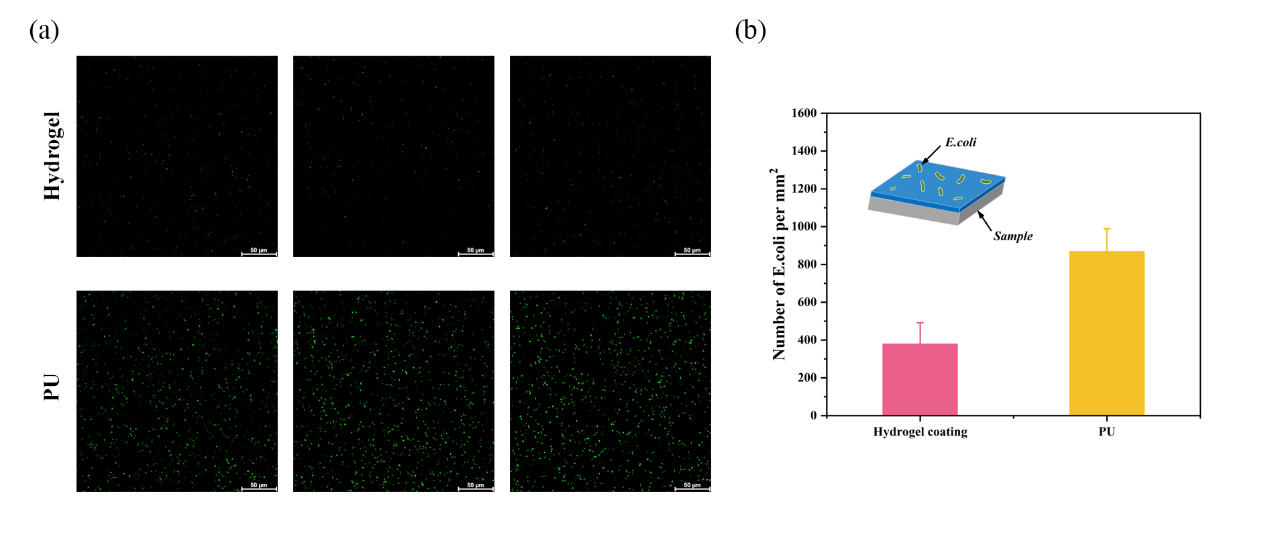


**Figure S13.** **Antifouling properties of hydrogel coating.** (a) Laser confocal microscope images of E. coli adhered to PU and PU with hydrogel coating after 24 h incubation. (b) The number of adhered E. coli per unit area (mm^2^) for each substrate (n = 3 repeats).

**
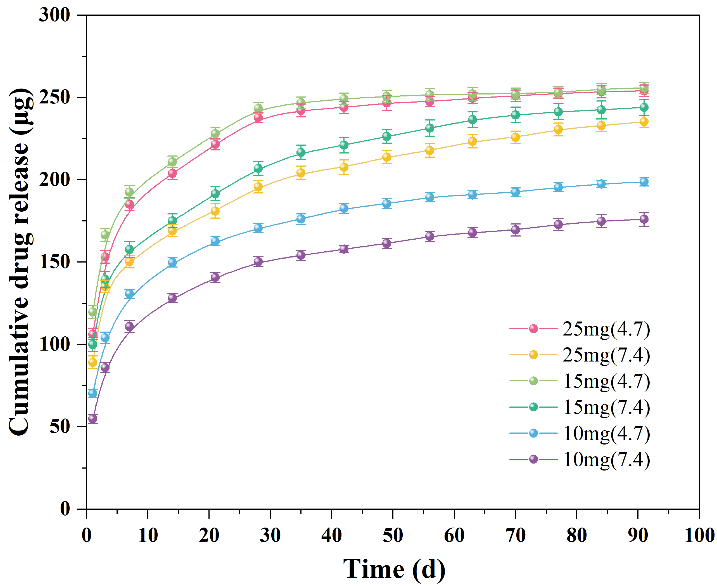
**

**Figure S14.** **In vitro cumulative release curves of the NPs-hydrogel coated ureteral stent with different concentrations of PFD-fPLGA NPs.** Hydrogel solutions with varying nanoparticle concentrations (10 mg/mL, 15 mg/mL, and 25 mg/mL) were prepared to fabricate NPs-hydrogel coated ureteral stents. Subsequently, drug release curves were characterized in vitro under different pH conditions.


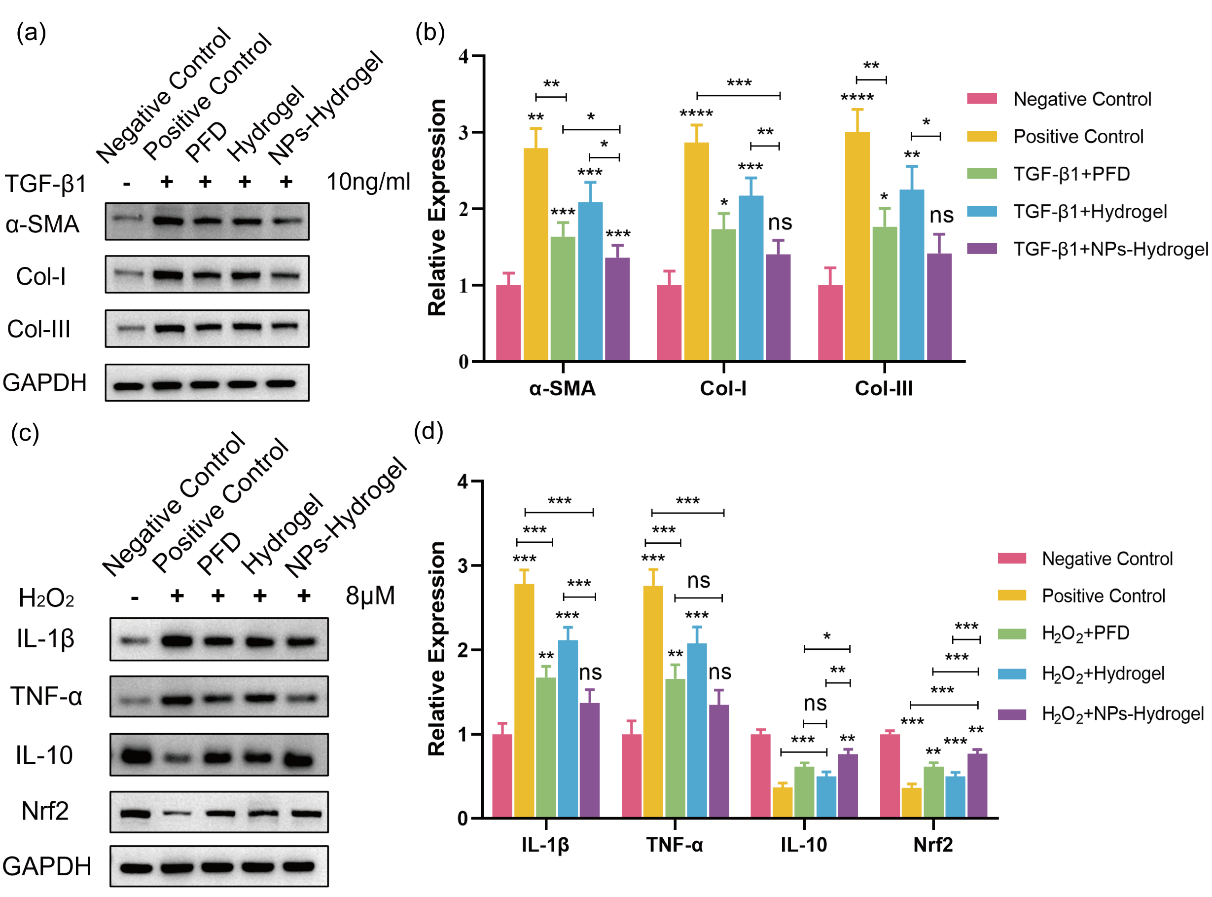


**Figure S15.** **Western blot results of proteins related to antifibrotic, anti-inflammatory, and antioxidant stress effects.** (a) Protein expression of α-SMA, Col-I, and Col-III in mouse fibroblasts with or without TGF-β1 treatment. (b) Protein expression of IL-1β, TNF-α, IL-10, and Nrf2 in mouse fibroblasts treated with or without H_2_O_2_ treatment. *P < 0.05, **P < 0.01, ***P < 0.001.

**
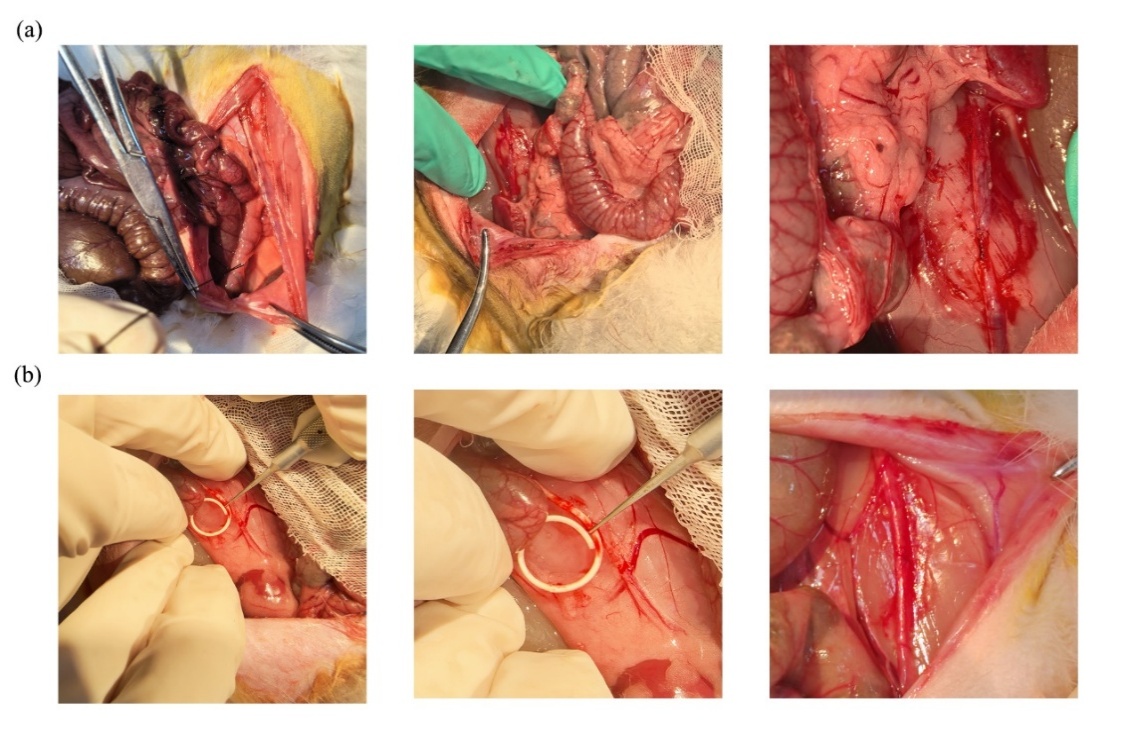
**

**Figure S16.** **Rabbit model of ureteral stricture.** (a) Establishment of rabbit ureteral stricture model; (b) Implantation of ureteral stent.

**
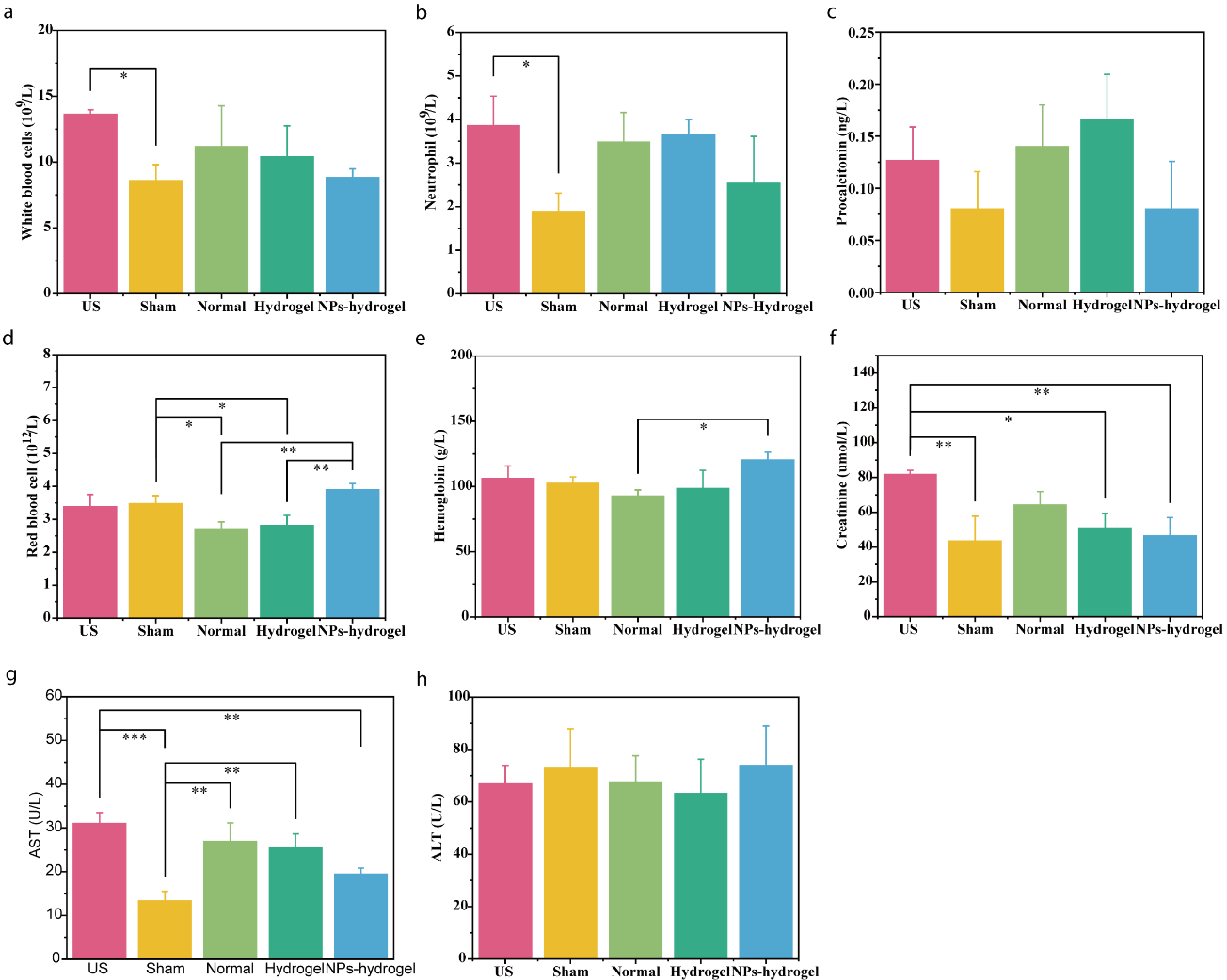
**

**Figure S17.** **Laboratory test results of animal experiments.** (a) White blood cell; (b) Neutrophil; (c) Procalcitonin; (d) Hemoglobin; (e) Red blood cell; (f) Creatinine; (g) Glutamic-oxalacetic transaminase; (h) Glutamic-pyruvic transaminase.


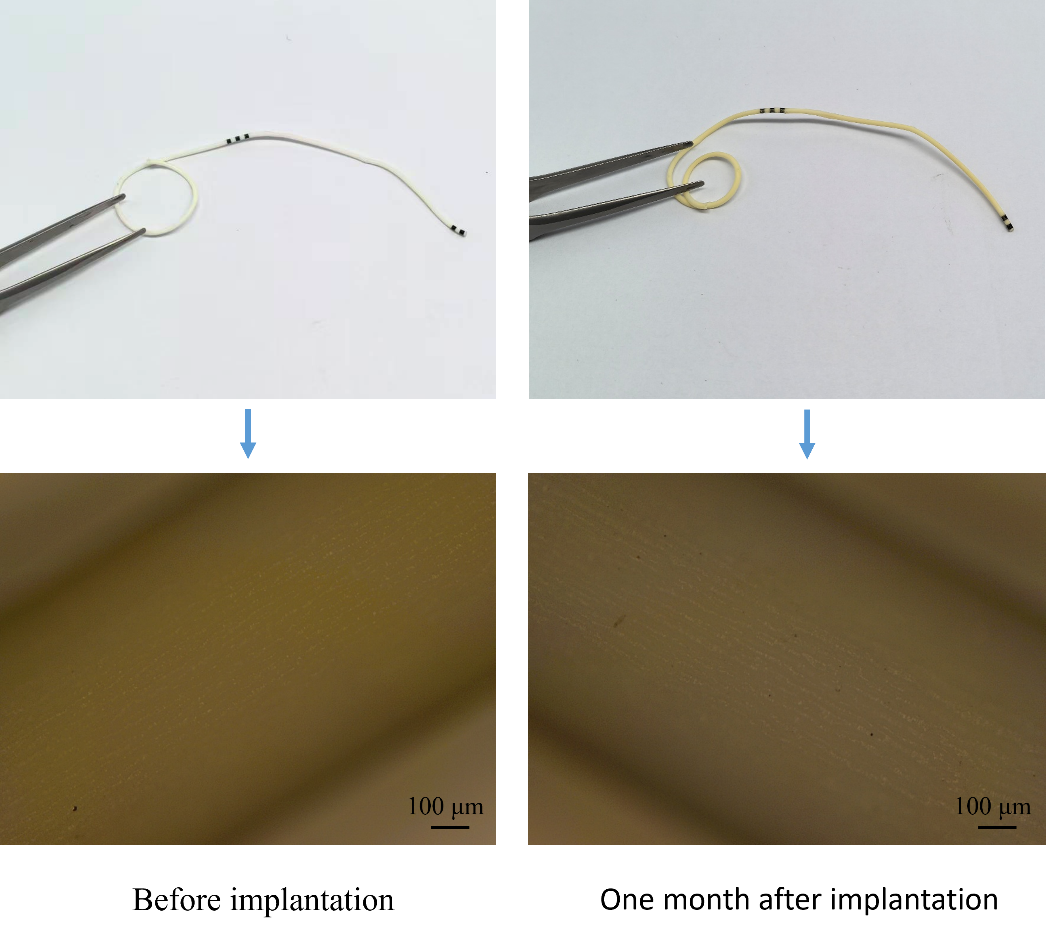


**Figure S18.** **Images of the stent before implantation into the body and after implantation**


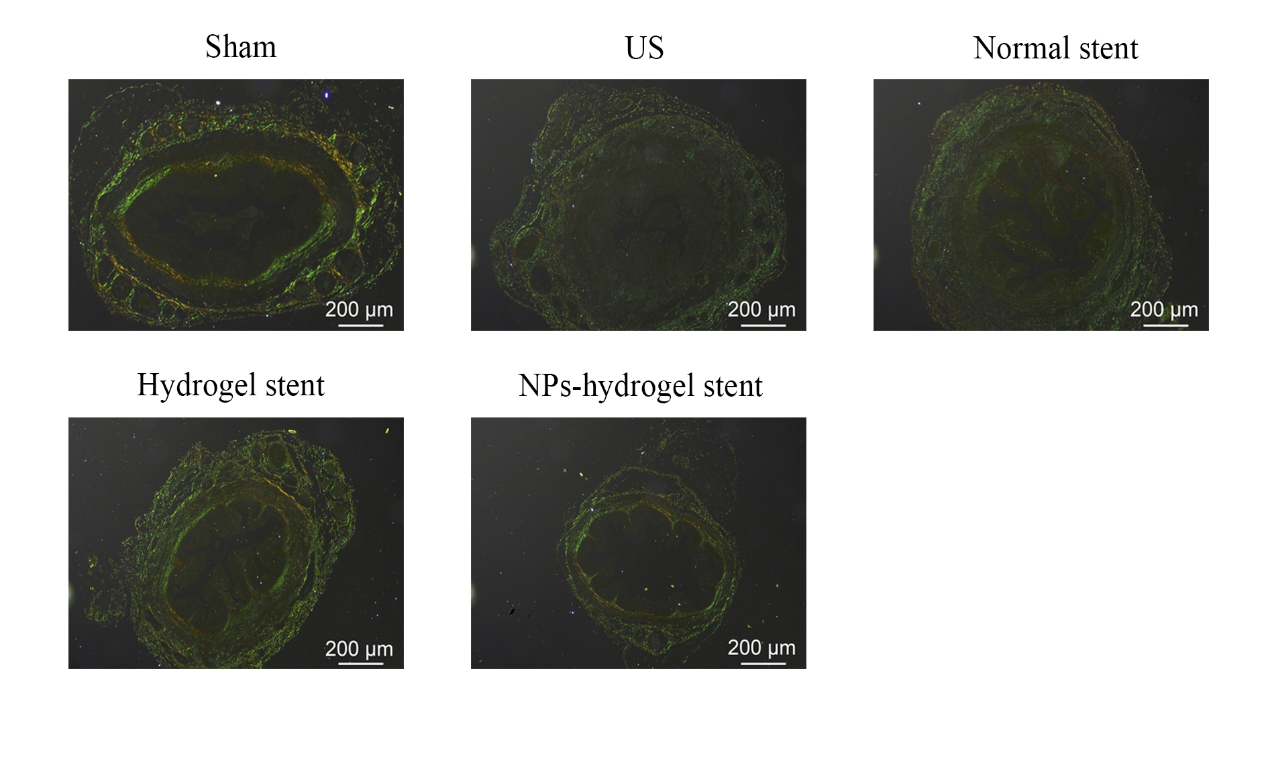


**Figure S19. Observe the collagen fibers in Picro Sirius Red stain sections using a polarizing light microscope.**
